# Upregulation of the long non-coding RNA CASC9 as a biomarker for squamous cell carcinoma

**DOI:** 10.1186/s12885-019-6021-6

**Published:** 2019-08-14

**Authors:** Madeleine Sassenberg, Johanna Droop, Wolfgang A. Schulz, Dimo Dietrich, Sophia Marie Loick, Constanze Wiek, Kathrin Scheckenbach, Nadine T. Gaisa, Michèle J. Hoffmann

**Affiliations:** 1 Department of Urology, Medical Faculty, Heinrich Heine University Duesseldorf, Moorenstr. 5, 40225 Duesseldorf, Germany; 2 Department of Otolaryngology, Head and Neck Surgery, University Hospital Bonn, Sigmund-Freud-Str. 25, 53105 Bonn, Germany; 3 Department of Otolaryngology, Medical Faculty, Heinrich Heine University Duesseldorf, Moorenstr. 5, 40225 Duesseldorf, Germany; 4 Institute for Pathology, University Hospital RWTH Aachen, Pauwelsstraße 30, 52074 Aachen, Germany

**Keywords:** Head-and-neck carcinoma, HNSCC, Squamous cell carcinoma, Biomarker, Long non-coding RNA, CASC9, HOTAIR, Bladder cancer

## Abstract

**Background:**

Few diagnostic and prognostic biomarkers are available for head-and-neck squamous cell carcinoma (HNSCC). Long non-coding RNAs (lncRNAs) have shown promise as biomarkers in other cancer types and in some cases functionally contribute to tumor development and progression. Here, we searched for lncRNAs useful as biomarkers in HNSCC.

**Methods:**

Public datasets were mined for lncRNA candidates. Two independent HNSCC tissue sets and a bladder cancer tissue set were analyzed by RT-qPCR. Effects of lncRNA overexpression or downregulation on cell proliferation, clonogenicity, migration and chemosensitivity were studied in HNSCC cell lines.

**Results:**

Data mining revealed prominently CASC9, a lncRNA significantly overexpressed in HNSCC tumor tissues according to the TCGA RNAseq data. Overexpression was confirmed by RT-qPCR analyses of patient tissues from two independent cohorts. CASC9 expression discriminated tumors from normal tissues with even higher specificity than HOTAIR, a lncRNA previously suggested as an HNSCC biomarker. Specificity of HNSCC detection by CASC9 was further improved by combination with HOTAIR. Analysis of TCGA pan-cancer data revealed significant overexpression of CASC9 across different other entities including bladder, liver, lung and stomach cancers and especially in squamous cell carcinoma (SCC) of the lung. By RT-qPCR analysis we furthermore detected stronger CASC9 overexpression in pure SCC of the urinary bladder and mixed urothelial carcinoma with squamous differentiation than in pure urothelial carcinomas. Thus, CASC9 might represent a general diagnostic biomarker and particularly for SCCs. Unexpectedly, up- or downregulation of CASC9 expression in HNSCC cell lines with low or high CASC9 expression, respectively, did not result in significant changes of cell viability, clonogenicity, migration or chemosensitivity.

**Conclusions:**

CASC9 is a promising biomarker for HNSCC detection. While regularly overexpressed, however, this lncRNA does not seem to act as a major driver of development or progression in this tumor.

**Electronic supplementary material:**

The online version of this article (10.1186/s12885-019-6021-6) contains supplementary material, which is available to authorized users.

## Background

Long non-coding RNAs (lncRNAs) have moved into the focus of cancer research as good candidates for tumor biomarkers and as regulators of various neoplastic cell properties. In general, lncRNAs are defined as being longer than 200 nucleotides and lacking a functional open reading frame. Apart from this general definition, they are highly diverse in structure and function. Many lncRNAs resemble mRNAs in being spliced, poly-adenylated and located in the cytoplasm. Some lncRNAs (referred to as long intergenic non-coding RNAs, lincRNAs) are transcribed from separate loci, whereas others are transcribed divergently from promoters of protein-coding genes or in antisense direction to these. A number of lncRNAs have been shown to regulate cellular processes including proliferation, apoptosis and differentiation in diverse physiological and pathological contexts [1]. Importantly, many lncRNAs are expressed in a cell type-specific manner and their expression changes during tumorigenesis. Dysregulation of lncRNA expression has been reported for different cancer types and may contribute to tumor development and progression [2, 3]. Prominent examples of such lncRNAs are TINCR, which contributes to keratinocyte differentiation [4], and HOTAIR, which is overexpressed in different cancer types, including head- and -neck squamous cell carcinoma (HNSCC) [5], and is typically associated with increased proliferation and migration of tumor cells.

HNSCC is a common malignancy caused mostly by exposure to carcinogens from cigarette smoking and alcohol consumption, or alternatively, by tumorigenic strains of human papillomavirus (HPV). Radiation therapy, surgery, chemotherapy, therapy with EGFR antibodies, immune checkpoint inhibitors or combined treatments are used for primary tumors and recurrent or metastatic disease. Patients with localized HNSCC and low tumor stage have a high chance of cure. Recurrent disease appears in up to 50% of the cases. High stage, metastatic and recurrent HNSCC have limited treatment options and therefore an unfavorable outcome [6]. To date, clinically validated prognostic biomarkers for HNSCC are lacking except for HPV positivity, which predicts favorable survival and better response to radio (chemo)-therapy [7]. Furthermore, diagnostic biomarkers to better discriminate precancerous mucosal lesions are desirable.

A large number of studies have investigated the expression of various microRNAs in HNSCC as potential biomarkers [8]. In contrast, few studies on lncRNAs in HNSCC are available to date [9]. We have therefore attempted to identify lncRNAs that are overexpressed in HNSCC and might serve as diagnostic and ideally also prognostic biomarkers. Data mining revealed several candidates. Here, we report the results of data mining and validation experiments for the most prominent candidate, CASC9. CASC9 (Cancer susceptibility candidate 9) located on chromosome 8q21.11 was first described [10] as an esophageal squamous cell carcinoma (ESCC)-associated lncRNA with increased expression in ESCC, comparable to HOTAIR in ESCC. Overexpression in ESCC was confirmed by additional studies [11, 12]. Expression was particularly upregulated in advanced stages and correlated with tumor size and poor overall survival suggesting CASC9 as a biomarker for ESCC diagnosis and prognosis.

We validated CASC9 overexpression in two independent HNSCC tissue sets by RT-qPCR and further investigated CASC9 expression in various other cancers. Finally, we performed in vitro experiments to explore the effect of CASC9 expression on cell proliferation, clonogenicity, migration or chemosensitivity. We found that CASC9 is upregulated in many HNSCC cases, particularly in late stages and tumors with extracapsular spread. A pan-cancer analysis revealed that CASC9 is also strongly overexpressed in different other entities including bladder, liver, lung and stomach cancers and especially in squamous cell carcinoma (SCC) of the lung. Analysis of a further tissue set comprising bladder cancers with different histologies by RT-qPCR demonstrated CASC9 overexpression predominantly in urothelial carcinomas with squamous differentiation or pure squamous bladder cancers. Collectively, these findings indicate CASC9 as a valuable diagnostic marker particularly for HNSCC and other squamous cell carcinomas. Discrimination between HNSCC tumor and non-cancerous tissues may be further improved by combination with HOTAIR detection, whose upregulation in HNSCC was confirmed in our study. However, since experimental modulation of CASC9 expression in HNSCC cell lines did not appear to exert a major influence on tumor cell properties in vitro, CASC9 may not be crucially involved in the establishment of the neoplastic phenotype in all HNSCC tumors, but may reflect the transformed state.

## Methods

### Patients and tissues

The Duesseldorf set of HNSCC tissue samples (DUS) used for quantitative real time RT-PCR analysis (RT-qPCR) comprised 32 tumor and 12 normal adjacent tissues, median patient age was 64.5 years. Six tumors were staged according to TNM version 7 as pT1, 13 as pT2, 6 as pT3, and 7 as pT4, six tumors were HPV positive determined by immunohistochemistry for p16^INK4A^. Information about p16^INK4A^ state was missing for six patients. Median follow-up time for this cohort was 43.6 months. The Bonn HNSCC cohort (BN) consisted of 79 patients. Expression data was obtained from 66 tumor and 56 normal adjacent tissues. Median age was 62 years. Eleven tumors were categorized as pT1, 33 as pT2, 24 as pT3, 10 as pT4; pT category of one tumor was unknown. Median follow-up time for the complete cohort was 48.0 months. HPV status of the BN cohort was determined using the HPV 3.5 LCD-Array Kit (Chipron GmbH, Berlin, Germany).

Both tissue sets were collected according to the principles expressed in the Declaration of Helsinki and with written patient informed consent as approved by the ethics committees of the medical faculties of the Heinrich Heine University Duesseldorf (study number 4698) and Friedrich Wilhelms University Bonn (Nr. 187/16), Germany.

A bladder cancer tissue set comprising 11 muscle-invasive pure urothelial carcinomas without any histological signs of squamous differentiation (UC), nine mixed tumors consisting of muscle-invasive urothelial carcinomas displaying histological areas with squamous differentiation patterns (MIX), 10 pure squamous carcinomas of the bladder (SCC) and 5 normal adjacent tissues were kindly provided by the RWTH centralized Biomaterial Bank Aachen (RWTH cBMB, Aachen, Germany) in accordance with the regulations of the biomaterial bank and the approval of the ethics committee of the medical faculty, RWTH Aachen (EK 206/09, study number 17).

The TCGA HNSCC dataset (http://cancergenome.nih.gov/) accessed via the TANRIC database (http://ibl.mdanderson.org/tanric/_design/basic/index.html) [13] consists of 426 tumor tissues and 41 normal adjacent tissues. This cohort comprised 27 patients with pT1, 128 with pT2, 117 with pT3, and 139 with pT4 tumors, 15 were of unknown pT category. HPV status provided by TCGA from 279 patients was determined by RNA-Seq data for the viral genes E6 and E7; with 36 patients HPV-positive and 243 HPV-negative [14]. Median age was 61 years. Median follow-up time for the complete cohort was 23.0 months.

### Cell lines

The HNSCC cell line panel consisted of UM (University of Michigan) -SCC 10A/ B, −11B, −14A/ B, −17A/ B, − 47, − 104 and UT (University of Turku) -SCC -14, − 24A/ B, − 33, as well as UD (University of Duesseldorf) -SCC 1, − 2, − 3, − 5, − 6, −7A, − 8, and FaDu. The suffixes A, B, and C indicate cell lines derived from primary tumor (A), metastatic (B) or recurrent (C) disease except for UD-SCC 7A, B and C which were derived from different sites of the same tumor, as described by Hoffmann et al. [15]. The immortalized keratinocyte cell line HaCaT was kindly provided by Dr. P. Boukamp, Duesseldorf [16]. Urothelial carcinoma cell lines (UCC) VM-CUB1, SW-1710, HT-1376, 5637, and BFTC-905 were obtained from the DSMZ (Braunschweig, Germany), other UCCs were kindly supplied by Dr. J. Fogh (New York, NY), Dr. M. A. Knowles (Leeds, UK) and Dr. B. Grossman (Houston, USA). Cell lines were verified by DNA fingerprint analysis and mycoplasm contamination was regularly checked.

Control cells comprised the spontaneously immortalized normal human urothelial cell line HBLAK [17] (kindly provided by CELLnTEC, Bern, Switzerland) and primary cultures of normal urothelial cells (UEC).

HNSCC and UCC lines were cultured in DMEM GlutaMAX-I (Gibco, Darmstadt, Germany) with 4.5 g/l D-glucose, pyruvate, and 10% FBS (Biochrom, Berlin, Germany). HBLAK cells were maintained in CnT-Prime Epithelial Culture Medium (CELLnTEC). Primary UEC cultures were established from fresh ureters and cultured in Epilife Medium (Gibco) as previously described (approved by the ethics committee of the medical faculty of the Heinrich Heine University Duesseldorf, study number 1788) [18]. All cells were cultured at 37 °C and 5% CO_2_.

To determine chemosensitivity of stably transfected HNSCC cell lines, cisplatin (Accord Healthcare, London, UK) was applied at indicated doses for 72 h.

### Lentiviral constructs for overexpression and knockdown of CASC9

For ectopic CASC9 expression the cDNA was cloned into the lentiviral expression vector pMF11bdEGNwo. SMARTvector lentiviral shRNA constructs (CASC9 #V3SH11246, nontargeting control #VSC11709) were purchased from Dharmacon (Lafayette, USA). Lentivirus production and cell transduction were performed as previously described [19, 20]. In brief, to produce replication-deficient lentiviruses, HEK-293 T cells were transfected with helper plasmid expression construct (pCD/NL-BH), envelope vector (pczVSV-G), and the target plasmids. Viral particles were harvested 48 h after transfection and used with 8 μg/ml polybrene to transduce cells (Sigma Aldrich, St. Louis, USA). Twenty-four hours after transduction, the supernatant containing viral particles was removed and the transduced cells were selected with neomycin (overexpression experiments) or puromycin (shRNA experiments). Stable overexpression and knockdown was confirmed by RT-qPCR.

### RNA isolation, cDNA synthesis and RT-qPCR

Total RNA was isolated using the Qiagen RNeasy Mini Kit (Qiagen, Hilden, Germany; DUS cohort) and NucleoSpin® RNA Kit (Macherey-Nagel GmbH, Dueren, Germany; BN cohort) according to the manufacturers’ protocols. RNA of non-epithelial control cells was kindly provided by Dr. C. Münk, (Heinrich Heine University Duesseldorf). For the DUS cohort, cDNA synthesis was performed with the QuantiTect Reverse Transcription Kit (Qiagen) with an extended incubation time of 30 min at 42 °C. For the BN cohort cDNA synthesis was performed using the SuperScript™ III First-Strand Synthesis System (Thermo Fisher Scientific, Waltham, MA, USA). QuantiTect SYBR Green RT-qPCR Kit (Qiagen) was used for RT-qPCR. Primer sequences for target genes and reference genes are listed in Additional file 1: Table S1. *TBP* (TATA-box binding protein) and *SDHA* (Succinate Dehydrogenase Complex Flavoprotein Subunit A) were measured as reference genes and a normalization factor was calculated for each sample using their geometric mean [21]. RT-qPCRs were run on the LightCycler 96 PCR platform (Roche, Penzberg, Germany).

### Measurements of cell viability, clonogenicity and migration

Cell viability was measured by MTT assay (Sigma-Aldrich, St. Louis, MO, USA). For colony formation assays cells were seeded at low density, maintained for 2 weeks and stained with Giemsa (Merck, Darmstadt, Germany) [22]. For wound healing assays cells were seeded into ibidi cell culture inserts (ibidi, Martinsried, Germany) until the cells reached confluency. Then, the culture insert was removed, the cells were washed with PBS, cultured in standard medium and photographic images were taken at given time points to evaluate scratch width.

### Database analysis and statistics

The TANRIC database was used to access publicly available RNA-Seq data for various tumor entities, especially for lncRNA expression. LncRNA expression values were obtained as log2 RPMK (reads per kilo base per million mapped reads). Cox *p*-values and log-rank p-values were also obtained from this database. Boxplots for pan-cancer analysis were created and Wilcoxon-rank-sum-test was calculated in R. *P*-values < 0.05 were considered statistically significant.

Further statistical analyses were conducted using SPSS, version 25 (SPSS Inc., Chicago, IL, USA). Comparisons of mean values were performed by Kruskal-Wallis (> 2 groups) and Wilcoxon-Mann-Whitney U (two groups) tests, respectively. Multiple pairwise comparisons between groups were tested by means of one-way analysis of variance (ANOVA) and post-hoc Bonferroni test. Correlations were calculated using Spearman’s rank correlation (Spearman’s ρ). Survival analyses were performed using the Kaplan-Meier method; *p*-values refer to log-rank test. For Kaplan-Meier analysis expression levels were dichotomized based on an optimized cut-off. Two-sided *P*-values < 0.05 were considered statistically significant. ROC-curves were created and AUC and best cutoff-values were calculated using the pROC-R-package [23].

## Results

To identify lncRNAs deregulated in HNSCC, we interrogated data published by Zou et al. [9] and public data from the TCGA consortium via the TANRIC database. Zou et al. identified 222 lncRNAs differentially expressed between HNSCC and normal control tissues. Analyzing TCGA data for these 222 candidates, we found 65 also significantly differentially expressed between tumor (*n* = 426) and normal (*n* = 41) tissues with altered expression correlating significantly with patient survival (Cox p-value and log-rank *p*-value < 0.01). We identified 14 lncRNAs with a median expression difference of at least 3-fold between tumor and normal tissues; 9 of these candidates were upregulated in cancers and 5 were downregulated (Table 1).

**Table 1:** Strongly differentially expressed lncRNAs in HNSCC tissues according to data published by Zou et al. [9]

Gene name UCSC genome browser	Chromosomal localisation	Size in bp	Alternative name	Neighbouring coding genes	lncRNA type	Expression in HNSCC tissues
ENSG00000233850.1	2q11.1	478	LNC-MRPS5–2	MAL	antisense	reduced
ENSG00000244128	3q26.1	563	LINC01322	SLITRK3	divergent	increased
ENSG00000248240.1	5p12	572	Lnc-NNT2	C5orf34	divergent	increased
ENSG00000152931	5q12.1	2316	PART1	PDE4D	antisense	reduced
ENSG00000249082	5q31.1	477	LOC340074	PITX1	intragenic	reduced
ENSG00000228789	6p21.33	1980	HCG 22	MUC22	intragenic	reduced
ENSG00000223485	6q27	647	LINC01615	THBS2	intragenic	increased
ENSG00000253187.2	7p15.2	1129	HOXA-AS4	HOXA10	antisense	increased
ENSG00000249395	8q21.11	1164	**CASC9**	HNF4G	intragenic	increased
ENSG00000235884.2	12p11.21	1645	LINC00941	CAPRIN2	intragenic	increased
ENSG00000244306	14q11.2	4264	LINC00516	POTEM	intragenic	increased
ENSG00000225210.4	14q11.2	4119	LINC01296	POTEM	intragenic	increased
ENSG00000258661.1	14q13.3	2613	NKX2–1-AS1	NKX2–1	antisense	reduced
ENSG00000184324.11	Xq28	847 b	CSAG3	MAGE-A2	intragenic	increased

CASC9 is bold printed as the main candidate investigated in this study and was identified by both data mining approaches

**Fig. 1:**
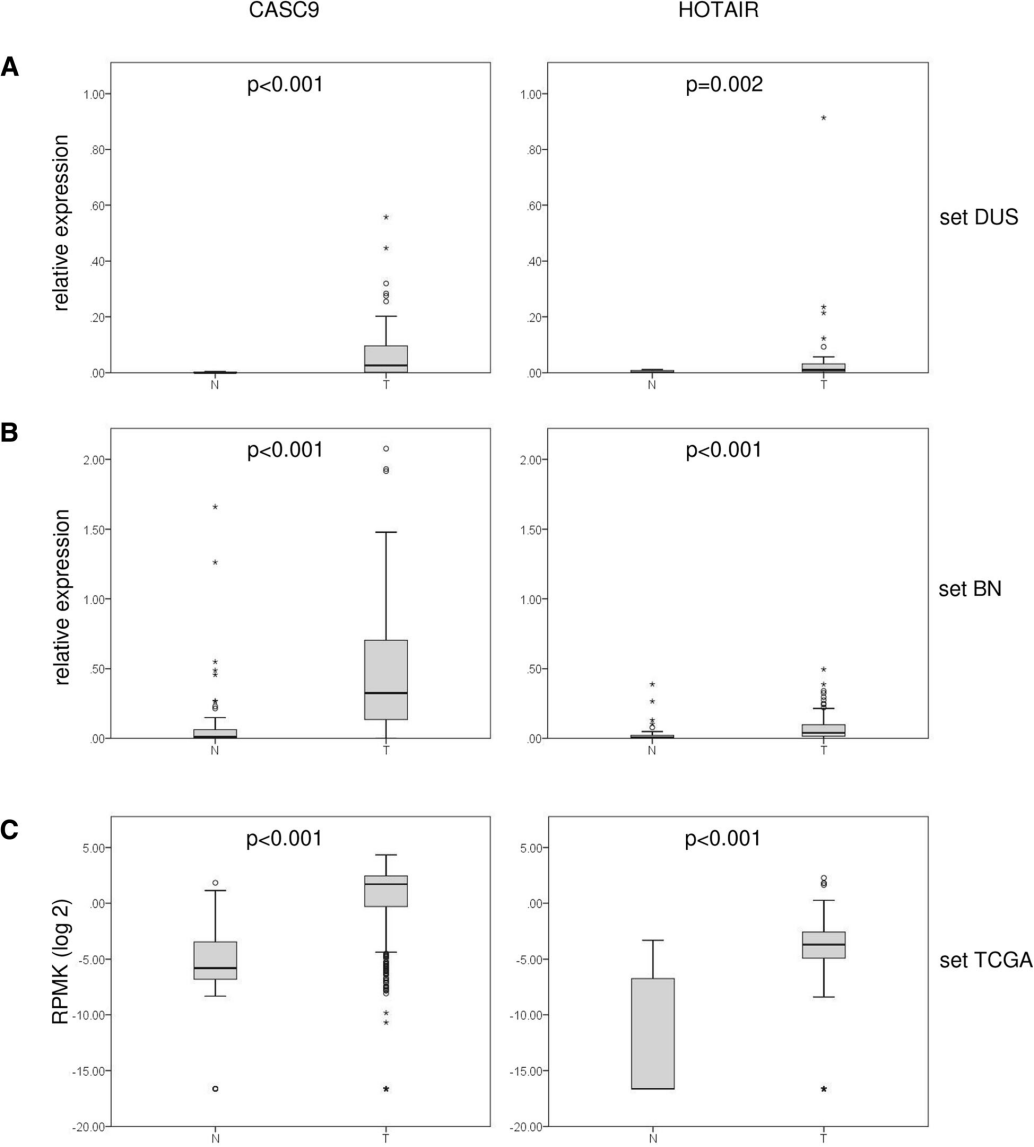
Expression of lncRNAs CASC9 and HOTAIR in different HNSCC tissue sets. Boxplot representations of lncRNA expression measured by RT-qPCR (relative expression to geometric mean of reference genes *SDHA* and *TBP*) in sets DUS (**a**) and BN (**b**) and by RNA-Seq in set TCGA (**c**) (public data from the TCGA HNSCC cancer cohort obtained from the TANRIC database; expression as log2 RPMK). *P*-values for difference between control (N) and tumor (T) samples were calculated by Mann-Whitney U-test

In a second approach, median expression in tumor and normal adjacent tissues was calculated for 38,184 lncRNAs from the extended provisional HNSCC TCGA dataset comprising 480 tumors and 42 normal adjacent tissue samples. As we sought robust biomarkers we selected those with at least 3-fold upregulation and at least RPKM median expression of 1 in tumors. This search revealed 20 candidates (Additional file 1: Table S2). CASC9, a lincRNA transcribed from a well-defined gene located on chromosome 8q21, was highlighted in both searches and was robustly expressed in RT-qPCR experiments using HNSCC tumor tissue samples (Fig. 1a), whereas other potential candidates were not unambiguously defined (e.g. POTEM) or yielded weak signals in RT-qPCR measurements (e.g. linc0116). For comparison, we instead included HOTAIR (Fig. 1a), which has been studied well in HNSCC [24] and in urothelial carcinoma [25].

In the TCGA training dataset, both lncRNAs were significantly upregulated (Fig. 1, *p* < 0.001, respectively). This upregulation was confirmed by RT-qPCR measurements in two tissue sample sets (Fig. 1 DUS and BN). In both sets, expression of CASC9 and HOTAIR was low in most normal tissues and often undetectable, but was strongly increased in most tumor samples. In the DUS set, CASC9 expression was higher in lower stage tumors (≤ pT2) and in older patients (Additional file 1: Table S3). Expression of HOTAIR was significantly lower in HPV-positive tumors. In the TCGA cohort HOTAIR expression was significantly increased in high grade tumors (*p* = 0.002) and associated with daily alcohol consumption (*p* = 0.011; Additional file 1: Table S4). High CASC9 expression was significantly associated with tumor localization (*p* < 0.001), high AJCC Stage (III and IV, *p* = 0.034) and extracapsular spread (*p* = 0.020). In the TCGA set expression of neither gene was associated with HPV status.

**Fig. 2:**
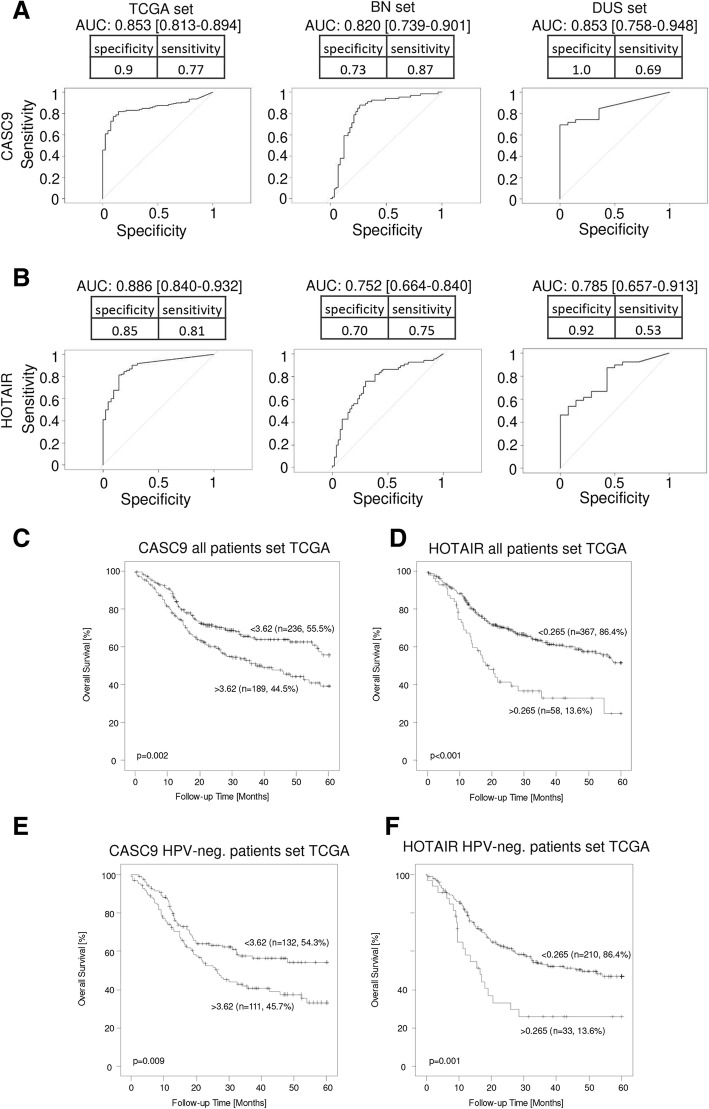
Diagnostic and prognostic power of CASC9 and HOTAIR in different HNSCC tissue sets. (**a**) Diagnostic power was determined by ROC curve analysis for CASC9 in the TCGA data set, the BN set and the DUS set and demonstrated excellent tumor specificity of CASC9. The same analysis was performed for lncRNA HOTAIR (**b**). 95% confidence interval values are given in brackets. Prognostic power was determined by Kaplan-Meier analysis. Increased expression of CASC9 and HOTAIR had significant impact on overall survival of all patients from the TCGA set (**c**, **d**) and also among the HPV-negative patients (**e**, **f**)

According to ROC curve analysis, tumor specificity of CASC9 was excellent in the TCGA set, with an area under the curve (AUC) of 0.853 (Fig. 2a); for HOTAIR AUC was 0.886 (Fig. 2b). Similarly, high tumor specificity was indicated by ROC analysis of the BN set and the DUS set (CASC9 AUC: 0.820 BN, 0.853 DUS, Fig. 2a; HOTAIR AUC: 0.752 BN, 0.785 DUS, Fig. 2b). Combined overexpression of CASC9 and HOTAIR in the DUS set discriminated perfectly between normal and cancerous tissues, but detected fewer cancer samples (Additional file 1: Table S5). Thus, combined analysis of both lncRNAs can improve specificity for cancer detection to a specificity of 1.0, albeit with a diminished sensitivity of 0.48. Kaplan-Meier analysis for patients of the TCGA cohort additionally demonstrated prognostic power for both lncRNA candidates. Patients with high expression of either CASC9 (*p* = 0.002) or HOTAIR (*p* < 0.001) experienced poor overall survival (Fig. 2c, d). Similar results were obtained by exclusive analysis of HPV-negative patients (Fig. 2e, f).

**Fig. 3:**
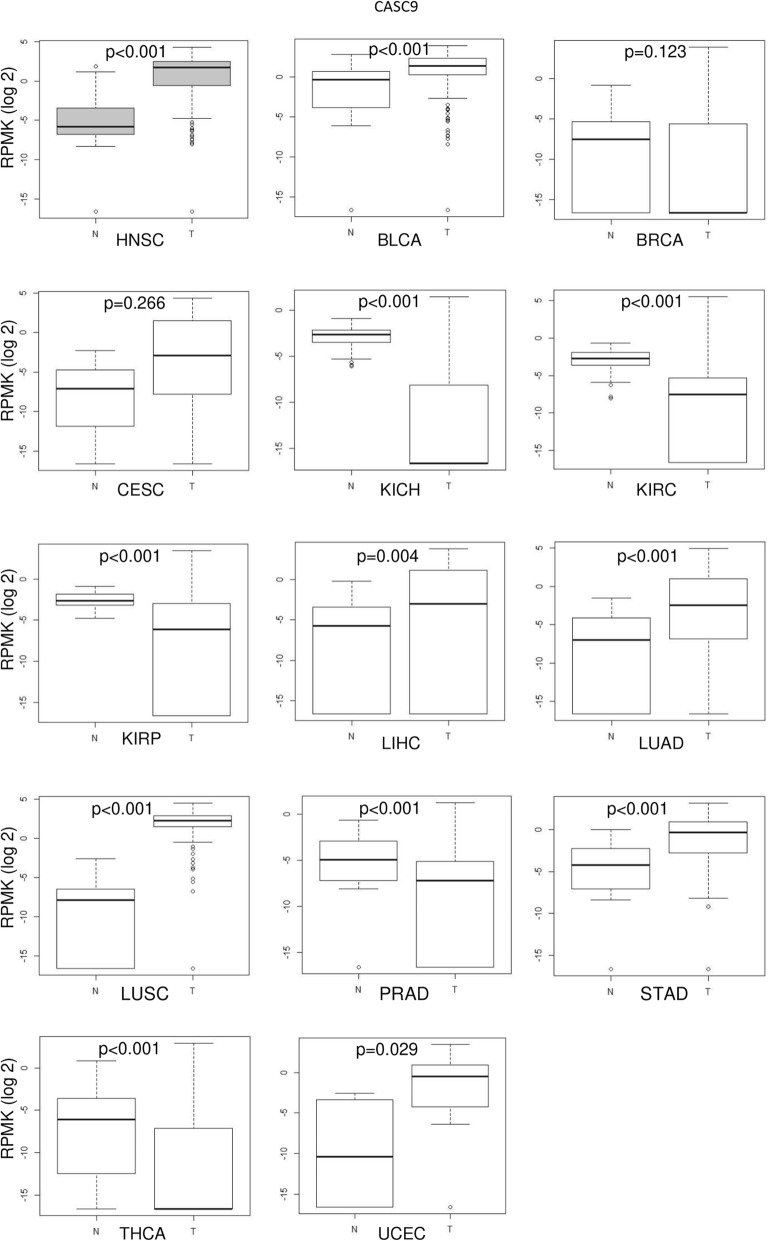
Pan-cancer analysis of CASC9 expression in TCGA datasets. The TANRIC database was used to access publicly available RNA-Seq data for CASC9 expression in various tumor entities: HNSC: head-neck squamous cell carcinoma; BLCA: urothelial bladder carcinoma; BRCA: breast invasive carcinoma; CESC: cervical squamous cell carcinoma and endocervical adenocarcinoma; KICH: kidney chromophobe; KIRC: kidney renal clear cell carcinoma; KIRP: kidney renal papillary cell carcinoma; LIHC: liver hepatocellular carcinoma; LUAD: lung adenocarcinoma; LUSC: lung squamous cell carcinoma.; PRAD: prostate adenocarcinoma; STAD: stomach adenocarcinoma; THCA: thyroid cancer; UCEC: uterine corpus endometrial carcinoma. LncRNA expression values were obtained as log2 RPMK (reads per kilo base per million mapped reads). Mann-Whitney U-test was applied to calculate *p*-values for differences between control (N) and tumour (T) samples

We further performed an in silico analysis of CASC9 expression in public pan-cancer TCGA data (Fig. 3). CASC9 was significantly overexpressed in cancers of various organs including bladder, liver, stomach and lung. Importantly, in addition to head- and -neck, CASC9 was also upregulated in squamous cell carcinomas from cervix and the lung, suggesting that strong CASC9 overexpression may be especially associated with aberrant squamous differentiation and may be valuable as a biomarker for cancer detection, but especially for squamous carcinomas.

**Fig. 4:**
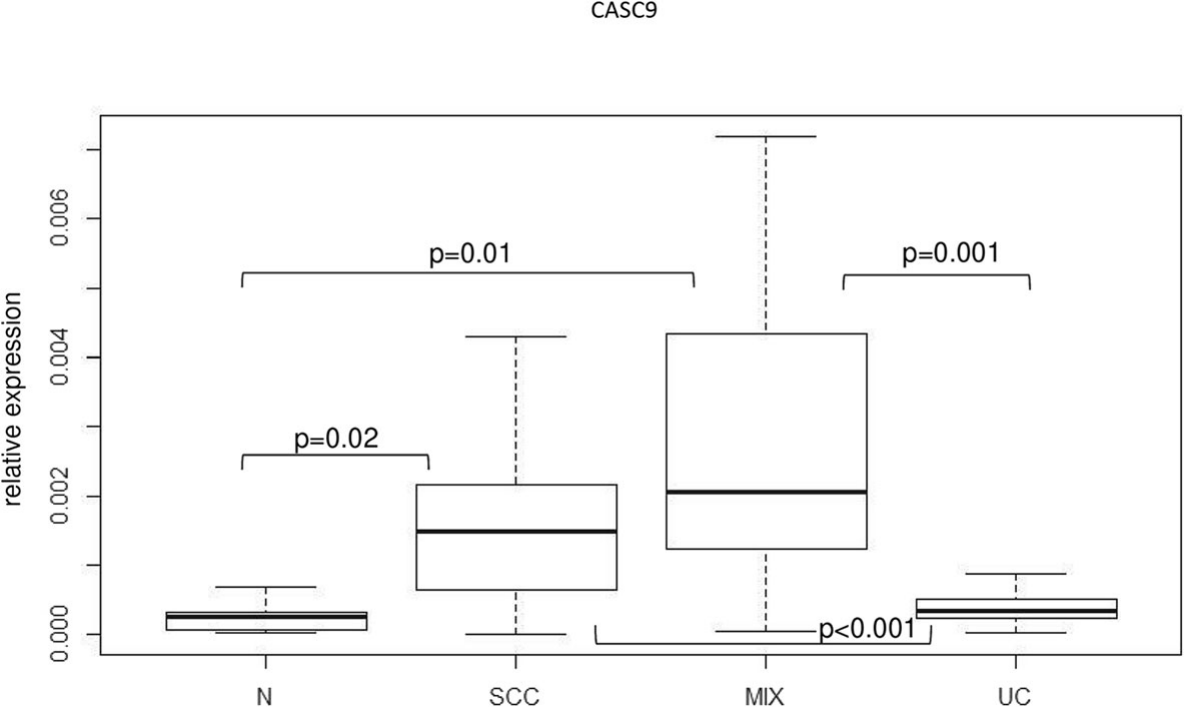
Expression of lncRNA CASC9 in different bladder cancer tissue specimen. Muscle-invasive urothelial carcinomas without any histological signs of squamous differentiation (UC) were compared with adjacent normal control samples (N), mixed tumors consisting of muscle-invasive urothelial carcinomas displaying histological areas with squamous differentiation (MIX) and pure squamous carcinomas of the bladder (SCC). LncRNA expression measured by RT-qPCR (relative expression to geometric mean of reference genes *SDHA* and *TBP*) is displayed as a boxplot graph. *P*-values for difference between control (N) and tumor samples were calculated by Wilcoxon-rank-sum-Test

To confirm this observation in an additional entity beyond HNSCC, we analyzed a set of bladder cancer tissues by RT-qPCR consisting of tumors with pure urothelial carcinoma (UC) histology, tumors with mixed urothelial and squamous cell carcinoma morphology (MIX), and nine specimens of pure squamous carcinoma of the bladder (SCC), which is a rare tumor type in industrialized countries. CASC9 was strongly increased in both MIX and SCC tumor tissues compared to morphologically pure UC and benign control tissues (Fig. 4). These results emphasize the strong relationship between highly elevated CASC9 expression and squamous differentiation.

In contrast, significant downregulation of CASC9 was found in the pan-cancer TCGA data in renal cell carcinoma (KIRC, KICH, KIRP), thyroid cancer (THCA), and prostate cancer (PRAD) (Fig. 3).

**Fig. 5:**
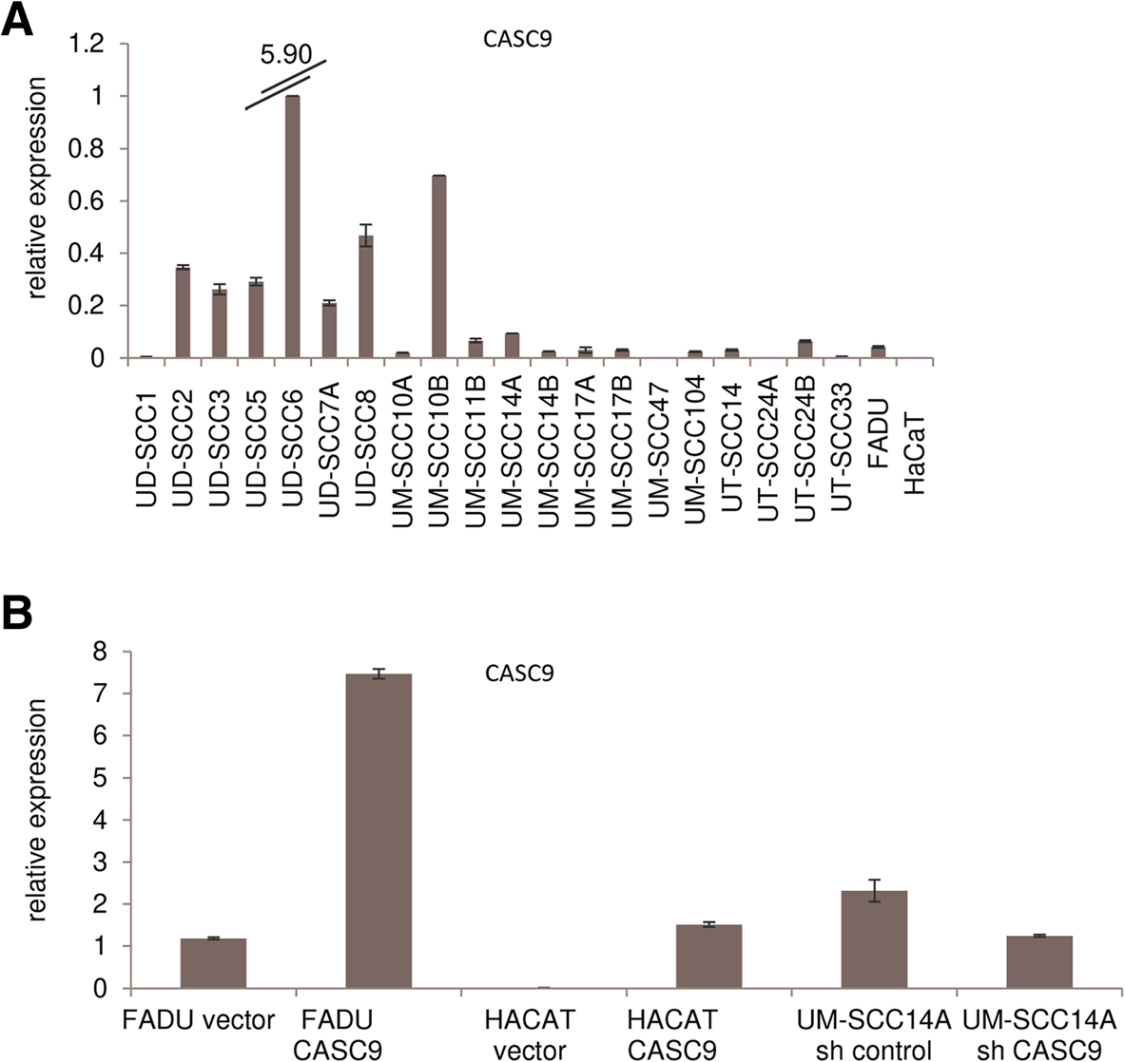
Expression of CASC9 in HNSCC cell lines. (**a**) Relative expression of CASC9 determined by RT-qPCR was heterogeneous across 21 HNSCC cell lines, but mostly increased compared to benign HaCat cells. (**b**) CASC9 overexpression and downregulation (sh) in stably transfected cells was validated by RT-qPCR

As a prerequisite for studying CASC9 function in HNSCC, we investigated CASC9 expression in cell lines from different cancer types by RT-qPCR. In accordance with the findings in tissues, CASC9 was expressed in 17 of 21 analyzed HNSCC cell lines, albeit at variable levels (Fig. 5a), but was almost undetectable in non-malignant HaCaT cells. Expression in UC cell lines varied across 16 cell lines (Additional file 2: Figure S1A). In keeping with the TCGA data, expression was very low in prostate cancer cell lines (Additional file 2: Figure S1B). Analysis of testicular cancer cell lines revealed overexpression in the embryonal carcinoma cell line NCCIT, but not in teratocarcinoma cell lines (Additional file 2: Figure S1C). We further measured CASC9 expression in cells found in the tumor microenvironment like mononucleated blood cells, macrophages, normal fibroblasts and cancer-associated fibroblasts. However, CASC9 expression was undetectable in all these cell types (data not shown), demonstrating exclusive cancer cell-specific expression.

**Fig. 6:**
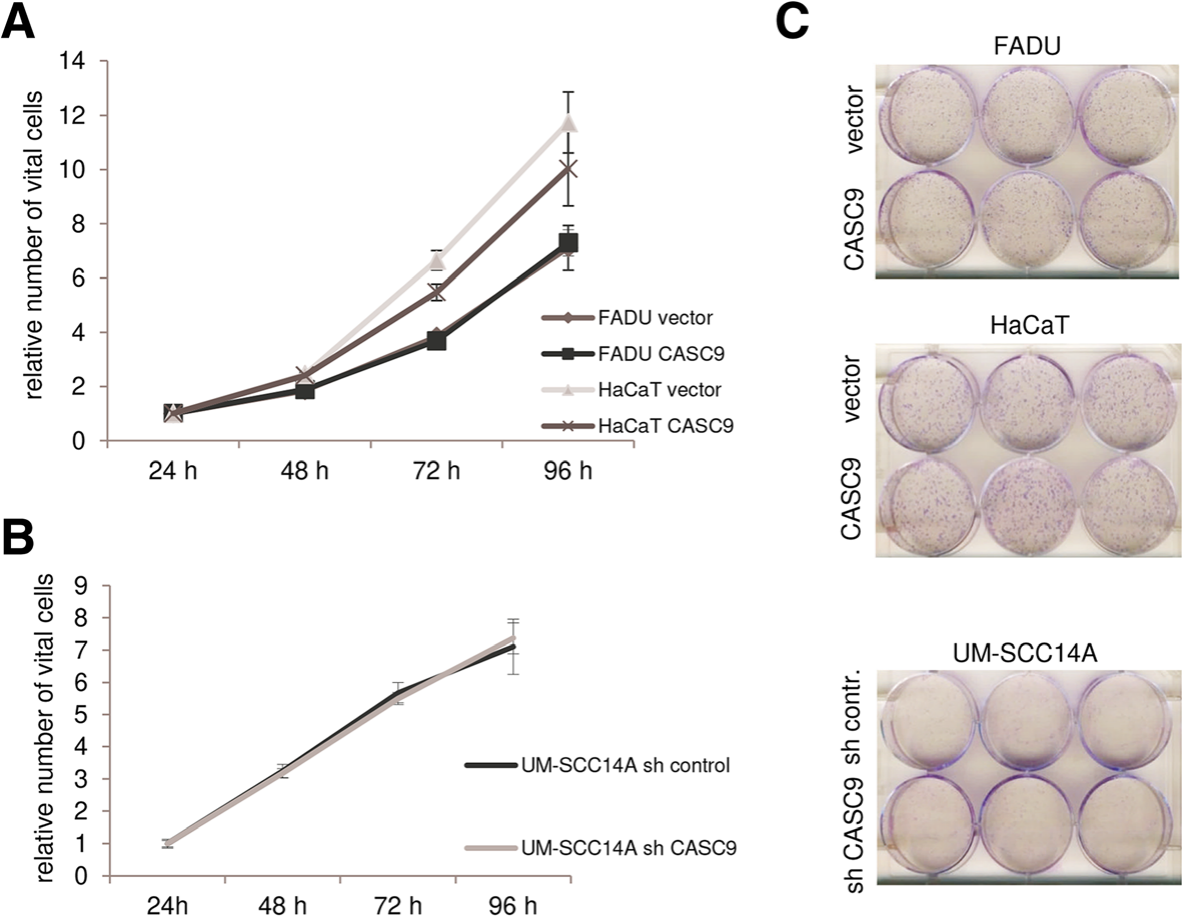
Effects of experimental CASC9 overexpression or downregulation on cell viability and clonogenicity. Effects of CASC9 overexpression (**a**) and downregulation (**b**) compared to controls on cell viability were measured by MTT assay for 96 h. (**c**) Colony formation capacity was visualized by Giemsa staining

**Fig. 7:**
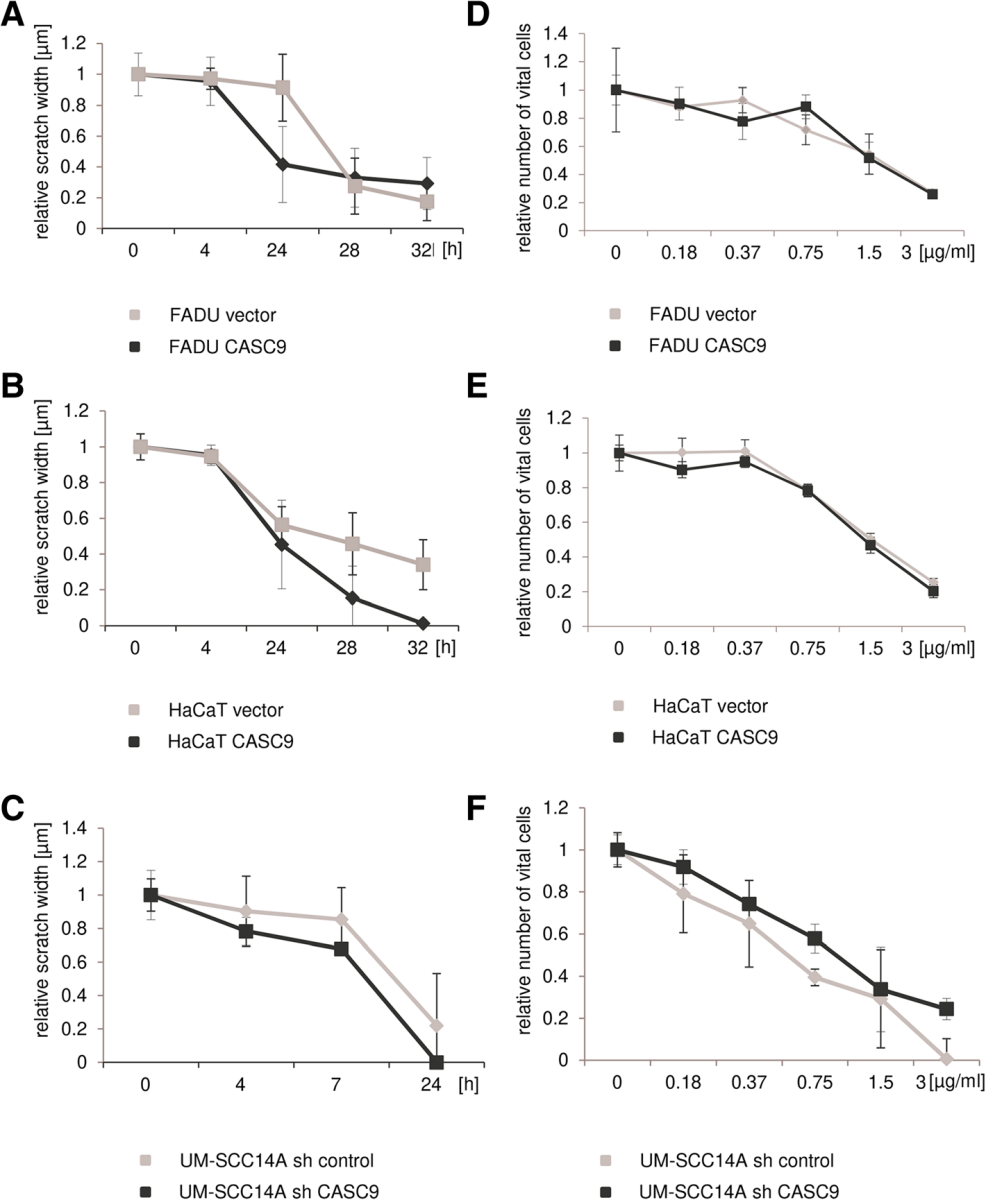
Effects of experimental CASC9 overexpression or downregulation on cell migration and chemosensitivity. Migration capacity of CASC9 overexpressing FADU (**a**) and HaCaT cells (**b**) and UM-SCC14A cells with CASC9 knockdown (**c**) compared to controls was measured by wound healing assay at given times points. Relative scratch width was normalized to 1 for the starting point of measurements. (**d-f**) Chemosensitivity was determined by MTT assay 72 h after treatment with the indicated doses of cisplatin

Finally, overexpressed CASC9 has been reported in recent publications to influence proliferation, migration and invasion of tumor cell lines from cancers of esophagus, lung, stomach, and liver [10–12, 26–29]. Association of CASC9 overexpression with chemoresistance was also observed [30]. To study these effects in HNSCC, we overexpressed CASC9 in non-malignant HaCaT cells and in HNSCC FADU cells, both with low endogenous expression. Conversely, a specific shRNA against CASC9 was stably expressed in UM-SCC-14A cells with high endogenous expression. Overexpression and downregulation of CASC9 were verified by RT-qPCR (Fig. 5b). None of these manipulations, however, resulted in significant changes in cell viability or clonogenicity (Fig. 6). Neither were significant changes observed in migration (Fig. 7a–c) and chemosensitivity towards cisplatin (Fig. 7d–f).

CASC9 has recently been reported to induce cell cycle arrest in ESCC cells by regulating the expression of the *PDCD4* gene [11]. *PCDC4* was heterogeneously expressed among HNSCC lines (Additional file 2: Figure S2A) and its expression was rather diminished in most UCC compared to benign controls (Additional file 2: Figure S2B). Neither CASC9 overexpression nor knockdown significantly affected *PDCD4* expression (Additional file 2: Figure S2C). Further reported CASC9 target genes were *CDK4*, CyclinD1 (*CCND1*), E-Cadherin (*CDH1*) and *BCL2* in lung adenocarcinoma [26], ESCC cells [12], oral squamous cell carcinoma [31] and in breast cancer [32]. However, neither of these genes displayed significant changes in expression according to analysis by RT-qPCR following experimental modulation of CASC9 in HNSCC cells. Moreover, across our panel of 21 HNSCC cell lines no correlations were observed between CASC9 and *CDK4* or Cyclin D1 and only weak correlations for E-Cadherin (Pearson *r* = 0.48) and BCL2 (Pearson *r* = 0.50) (Additional file 2: Figure S3A-D).

## Discussion

Investigation of tumor-related lncRNAs may provide novel cancer biomarkers, in particular for malignancies like HNSCC where genomic characterization has not yet yielded significant improvements in diagnostics and prognostication. We therefore sought to identify lncRNAs overexpressed in HNSCC that might serve as diagnostic and ideally also prognostic biomarkers by data mining of public data and validation experiments.

To identify new candidates suitable as biomarkers we searched for lncRNAs that were robustly overexpressed in HNSCC and associated with patient outcome. Comparison of candidates from two large studies [9, 14] ultimately yielded several candidate lncRNAs which were substantially upregulated in cancers according to RNA-Seq. However, several candidates were not unambiguously defined or yielded weak signals in RT-qPCR measurements in HNSCC tissues. This observation is not unexpected, as lncRNA genes are more difficult to annotate and generally more weakly transcribed than protein-coding genes. We therefore focused on CASC9 which was retrieved by both searches and robustly expressed in tumors according to RT-qPCR.

CASC9 was first described as an esophageal squamous cell carcinoma (ESCC)-associated lncRNA with increased expression in ESCC comparable to HOTAIR. Overexpression in ESCC was confirmed by additional studies [10–12]. Upregulated expression was associated with advanced stages, tumor size and poor overall survival suggesting CASC9 as a biomarker for ESCC diagnosis and prognosis.

Our results in HNSCC samples obtained by RT-qPCR analysis of two different patient cohorts and by in silico analysis of public TCGA data accord well with those in ESCC, likewise indicating a high diagnostic potential. CASC9 expression had excellent tumor specificity according to ROC curve analysis, comparable to results reported in ESCC [12], and high CASC9 expression was significantly associated with high AJCC stage and extracapsular spread, indicating further diagnostic power. Although suggested by the TCGA data, a prognostic value for CASC9 could not be confirmed by RT-qPCR results in our own cohorts. This difference may relate to the different representation of tumor stages and localizations between the TCGA and our tissue sets. Evidently, further analysis of larger cohorts with specific assays is required to address this issue.

In adenocarcinomas of the lung increased CASC9 expression was also associated with tumor size, stage, lymph node metastasis and a poor prognosis [26]. Similar results were reported for gastric cancer, where CASC9 was also highly expressed in chemoresistant cell lines, and for hepatocellular carcinoma [28, 29]. Nevertheless, our analysis of TCGA pan-cancer data and various cell lines indicated that CASC9 is upregulated across cancers of various organs and in further squamous carcinomas. CASC9 may therefore be useful as a general diagnostic biomarker for cancers and particularly squamous cell carcinomas of different organs, like esophagus, head- and -neck, cervix and lung. CASC9 was also significantly overexpressed in many bladder cancer specimen, particularly in urothelial cancers with squamous differentiation (MIX) and especially strongly in pure squamous carcinoma of the bladder (SCC).

The specificity and sensitivity of HNSCC detection by CASC9 may be improved by combination with further lncRNAs in a biomarker panel [33], such as HOTAIR, as observed in our study. Our study confirms the previously reported upregulation of HOTAIR, particularly in high grade HNSCC [5]. HOTAIR originates from the *HOXC* locus on chromosome 12q13.3 and regulates expression of homeotic HOX loci, but also of many other genes [25]. HOTAIR upregulation is associated with poor patient prognosis in mammary and esophageal squamous cell carcinoma [34, 35] and other malignancies [36]. These findings extend to HNSCC. Thus, the suitability of CASC9 as a prognostic biomarker requires further study, but a considerably body of concurrent data indicates CASC9 as a strong diagnostic marker for squamous cancers. Its specificity may be even further increased by other lncRNA biomarkers like HOTAIR.

Since previous studies with cell lines from other cancer types reported stimulating effects of CASC9 expression on proliferation, migration and invasion or inhibitory effects on apoptosis [10–12, 26–29], we modulated CASC9 expression by either stable overexpression or shRNA-mediated knock down in suitable HNSCC cell lines and in the benign HaCaT cell line. Surprisingly, in the light of the previous reports, we did not observe robust changes in cell proliferation, clonogenicity or migration capacity in our analyses. We note, however, that the effects reported in studies in other cancer types ranged from subtle to profound. Likewise, the mechanisms by which CASC9 exerted its pro-neoplastic effects were also highly diverse among previous studies. For instance, in ESCC, the stimulatory effects of CASC9 on proliferation and the cell cycle have been ascribed to recruitment of the histone methyltransferase EZH2 which then downregulates the pro-apoptotic protein PDCD4 [11]. In HNSCC lines *PDCD4* was heterogeneously expressed and was unaffected by CASC9 modulation, indicating that the mechanism discovered in ESCC does not seem to be relevant in HNSCC cells. Similarly, other reported downstream target genes like *CDK4, CCND1*, *CDH1* and *BCL2* did not respond to up- or downregulation of CASC9 in HNSCC cells and only *BCL2* and *E-Cadherin (CDH1)* expression correlated moderately with CASC9 expression across our cell line panel, indicating that they may be rather coregulated by common underlying mechanisms.

Collectively, these observations suggest that upregulation of CASC9 is common in HNSCC and other, especially squamous cancers. The functional contribution of CASC9 to the neoplastic phenotype may be highly variable and appears to strongly depend on context, which is a typical property of lncRNAs. We therefore think it is unlikely that CASC9 is a general major driver of tumor development or progression. Obviously, its upregulation could also constitute a bystander effect of carcinogenesis and particularly of aberrant squamous differentiation in some tumors. Notably, this does not preclude using CASC9 as a valuable biomarker of HNSCC, as a diagnostic biomarker does not necessarily have to be functionally important. For example, PSA/KLK3 is generally used for detection, prognosis and monitoring in prostate cancer, but has at most marginal effects on tumor growth.

## Conclusions

CASC9 is robustly overexpressed in HNSCC, a promising candidate for tumor detection, and potentially of squamous cell carcinomas in all organs. Our data suggest that although CASC9 is an excellent indicator of cancer in the oropharyngeal tract, it may not be crucially involved in the establishment of the neoplastic phenotype in all cases. An important question for future work is therefore which factors drive the overexpression of CASC9 in HNSCC and other squamous cell carcinomas.

## Additional files

Additional file 1: **Table S1.** Oligonucleotide sequences for named targets. Forward and reverse primer sequences are given in 5′ to 3’orientation. **Table S2.** Strongly overexpressed lncRNAs in HNSCC tissues with potential prognostic value according to TCGA data. **Table S3.** Clinical and histopathological parameters of tissue set DUS. **Table S4.** Clinical and histopathological parameters of the TCGA cohort, * TNM, 4th–7th Edition. **Table S5.** Combined analysis of specificity and sensitivity for CASC9 and HOTAIR in the DUS HNSCC tissue sample set [37]. (DOC 255 kb)

Additional file 2: **Figure S1.** Expression of CASC9 in cell lines of other tumor entities. (**a**) Relative expression of CASC9 was determined by RT-qPCR across 16 urothelial carcinoma cell lines compared to the benign urothelial control cell line HBLAK and a primary urothelial cell culture (UEC). (**b**) Relative expression of CASC9 in prostate cancer cell lines, benign control cells (PREC, PNT-2, BPH-1) as well as normal (N) and cancerous (T) tissue. (**c**) Relative expression of CASC9 in testicular cancer cell lines compared to normal (N) and cancerous (T) tissue. **Figure S2.** Expression of a putative downstream target gene *PDCD4* in HNSCC and UCC cell lines. (**a**) Relative expression of PDCD4 mRNA was determined by RT-qPCR across 21 HNSCC cell lines compared to benign HaCat cells. (**b**) Relative *PDCD4* expression across 16 urothelial carcinoma cell lines compared to the benign urothelial control cell line HBLAK and a primary urothelial cell culture (UEC). (**c**) Relative expression of *PDCD4* in cells with CASC9 overexpression or downregulation (sh). No significant changes were observed. **Figure S3.** Expression of a putative downstream target genes *CDK4*, *CCND1, CDH1* and *BCL2* in HNSCC cell lines. (**a**) Relative expression of CDK4 mRNA was determined by RT-qPCR in cell lines following CASC9 modulation and across 21 HNSCC cell lines compared to benign HaCat cells. (**b**) Relative expression of CCND1 mRNA was determined by RT-qPCR in cell lines following CASC9 modulation and across 21 HNSCC cell lines compared to benign HaCat cells. (**c**) Relative expression of E-Cadherin mRNA was determined by RT-qPCR in cell lines following CASC9 modulation and across 21 HNSCC cell lines compared to benign HaCat cells. (**d**) Relative expression of BCL2 mRNA was determined by RT-qPCR in cell lines following CASC9 modulation and across 21 HNSCC cell lines compared to benign HaCat cells. (ZIP 1403 kb)

## Data Availability

The datasets used and analysed in the current study are available from the corresponding author on reasonable request.

## Abbreviations

AUC: Area under the curve
BN: Tissue set from Bonn
CAM: Chicken chorioallantoic membrane model
DUS: Tissue set from Duesseldorf
ESCC: Esophageal squamous cell carcinoma
HNSCC: Head-and-neck carcinoma
HPV: Human papillomavirus
KIRC, KICH, KIRP: Renal carcinoma
lncRNA: Long non-coding RNA
MIX: Urothelial carcinoma with squamous differentiation
PRAD: Prostate cancer
RPKM: Reads per kilobase million
RT-qPCR: Quantitative real time RT-PCR analysis
SCC: Squamous cell carcinoma
THCA: Thyroid cancer
UC: Urothelial carcinoma
UCC: Urothelial carcinoma cell lines

## References

[1] Fatica A, Bozzoni I (2014). Long non-coding RNAs: new players in cell differentiation and development. Nat Rev Genet.

[2] Dhamija S, Diederichs S (2016). From junk to master regulators of invasion: lncRNA functions in migration, EMT and metastasis. Int J Cancer.

[3] Haemmerle M, Gutschner T (2015). Long non-coding RNAs in cancer and development: where do we go from here?. Int J Mol Sci.

[4] Kretz M, Siprashvili Z, Chu C, Webster DE, Zehnder A, Qu K (2013). Control of somatic tissue differentiation by the long non-coding RNA TINCR. Nature..

[5] Troiano G, Caponio VCA, Boldrup L, Gu X, Muzio LL, Sgaramella N (2017). Expression of the long non-coding RNA HOTAIR as a prognostic factor in squamous cell carcinoma of the head and neck: a systematic review and meta-analysis. Oncotarget..

[6] Braakhuis BJ, Brakenhoff RH, Leemans CR (2012). Treatment choice for locally advanced head and neck cancers on the basis of risk factors: biological risk factors. Ann Oncol.

[7] Ang KK, Harris J, Wheeler R, Weber R, Rosenthal DI, Nguyen-Tan PF (2010). Human papillomavirus and survival of patients with oropharyngeal cancer. N Engl J Med.

[8] Sethi N, Wright A, Wood H, Rabbitts P (2014). MicroRNAs and head and neck cancer: reviewing the first decade of research. Eur J Cancer.

[9] Zou AE, Ku J, Honda TK, Yu V, Kuo SZ, Zheng H (2015). Transcriptome sequencing uncovers novel long noncoding and small nucleolar RNAs dysregulated in head and neck squamous cell carcinoma. RNA..

[10] Pan Z, Mao W, Bao Y, Zhang M, Su X, Xu X (2016). The long noncoding RNA CASC9 regulates migration and invasion in esophageal cancer. Cancer Med.

[11] Wu Y, Hu L, Liang Y, Li J, Wang K, Chen X (2017). Up-regulation of lncRNA CASC9 promotes esophageal squamous cell carcinoma growth by negatively regulating PDCD4 expression through EZH2. Mol Cancer.

[12] Gao GD, Liu XY, Lin Y, Liu HF, Zhang GJ (2018). LncRNA CASC9 promotes tumorigenesis by affecting EMT and predicts poor prognosis in esophageal squamous cell cancer. Eur Rev Med Pharmacol Sci.

[13] Li J, Han L, Roebuck P, Diao L, Liu L, Yuan Y (2015). TANRIC: an interactive open platform to explore the function of lncRNAs in Cancer. Cancer Res.

[14] Cancer Genome Atlas N (2015). Comprehensive genomic characterization of head and neck squamous cell carcinomas. Nature..

[15] Hoffmann TK, Sonkoly E, Hauser U, van Lierop A, Whiteside TL, Klussmann JP (2008). Alterations in the p53 pathway and their association with radio- and chemosensitivity in head and neck squamous cell carcinoma. Oral Oncol.

[16] Boukamp P, Petrussevska RT, Breitkreutz D, Hornung J, Markham A, Fusenig NE (1988). Normal keratinization in a spontaneously immortalized aneuploid human keratinocyte cell line. J Cell Biol.

[17] Hoffmann MJ, Koutsogiannouli E, Skowron MA, Pinkerneil M, Niegisch G, Brandt A (2016). The new immortalized Uroepithelial cell line HBLAK contains defined genetic aberrations typical of early stage urothelial tumors. Bladder Cancer.

[18] Swiatkowski S, Seifert HH, Steinhoff C, Prior A, Thievessen I, Schliess F (2003). Activities of MAP-kinase pathways in normal uroepithelial cells and urothelial carcinoma cell lines. Exp Cell Res.

[19] Kaletsch A, Pinkerneil M, Hoffmann MJ, Jaguva Vasudevan AA, Wang C, Hansen FK (2018). Effects of novel HDAC inhibitors on urothelial carcinoma cells. Clin Epigenetics.

[20] Schmidt EM, Wiek C, Parkinson OT, Roellecke K, Freund M, Gombert M (2015). Characterization of an additional splice acceptor site introduced into CYP4B1 in Hominoidae during evolution. PLoS One.

[21] Vandesompele J, De Preter K, Pattyn F, Poppe B, Van Roy N, De Paepe A (2002). Accurate normalization of real-time quantitative RT-PCR data by geometric averaging of multiple internal control genes. Genome Biol.

[22] Holscher AS, Schulz WA, Pinkerneil M, Niegisch G, Hoffmann MJ (2018). Combined inhibition of BET proteins and class I HDACs synergistically induces apoptosis in urothelial carcinoma cell lines. Clin Epigenetics.

[23] Robin X, Turck N, Hainard A, Tiberti N, Lisacek F, Sanchez JC (2011). pROC: an open-source package for R and S+ to analyze and compare ROC curves. BMC Bioinf.

[24] Sun S, Wu Y, Guo W, Yu F, Kong L, Ren Y (2018). STAT3/HOTAIR signaling Axis regulates HNSCC growth in an EZH2-dependent manner. Clin Cancer Res.

[25] Heubach J, Monsior J, Deenen R, Niegisch G, Szarvas T, Niedworok C (2015). The long noncoding RNA HOTAIR has tissue and cell type-dependent effects on HOX gene expression and phenotype of urothelial cancer cells. Mol Cancer.

[26] Zhou J, Xiao H, Yang X, Tian H, Xu Z, Zhong Y (2018). Long noncoding RNA CASC9.5 promotes the proliferation and metastasis of lung adenocarcinoma. Sci Rep.

[27] Liang Y, Chen X, Wu Y, Li J, Zhang S, Wang K (2018). LncRNA CASC9 promotes esophageal squamous cell carcinoma metastasis through upregulating LAMC2 expression by interacting with the CREB-binding protein. Cell Death Differ.

[28] Shang C, Sun L, Zhang J, Zhao B, Chen X, Xu H (2017). Silence of cancer susceptibility candidate 9 inhibits gastric cancer and reverses chemoresistance. Oncotarget..

[29] Klingenberg M, Gross M, Goyal A, Polycarpou-Schwarz M, Miersch T, Ernst AS (2018). The long noncoding RNA Cancer susceptibility 9 and RNA binding protein heterogeneous nuclear ribonucleoprotein L form a complex and Coregulate genes linked to AKT signaling. Hepatology..

[30] Ma P, Zhang M, Nie F, Huang Z, He J, Li W (2017). Transcriptome analysis of EGFR tyrosine kinase inhibitors resistance associated long noncoding RNA in non-small cell lung cancer. Biomed Pharmacother.

[31] Yang Y, Chen D, Liu H, Yang K (2019). Increased expression of lncRNA CASC9 promotes tumor progression by suppressing autophagy-mediated cell apoptosis via the AKT/mTOR pathway in oral squamous cell carcinoma. Cell Death Dis.

[32] Shao G, Wang M, Fan X, Zhong L, Wang Z, Zhang P (2019). lncRNA CASC9 positively regulates CHK1 to promote breast cancer cell proliferation and survival through sponging the miR195/497 cluster. Int J Oncol.

[33] Droop J, Szarvas T, Schulz WA, Niedworok C, Niegisch G, Scheckenbach K (2017). Diagnostic and prognostic value of long noncoding RNAs as biomarkers in urothelial carcinoma. PLoS One.

[34] Gupta RA, Shah N, Wang KC, Kim J, Horlings HM, Wong DJ (2010). Long non-coding RNA HOTAIR reprograms chromatin state to promote cancer metastasis. Nature..

[35] Lv XB, Lian GY, Wang HR, Song E, Yao H, Wang MH (2013). Long noncoding RNA HOTAIR is a prognostic marker for esophageal squamous cell carcinoma progression and survival. PLoS One.

[36] Botti G, Marra L, Malzone MG, Anniciello A, Botti C, Franco R (2017). LncRNA HOTAIR as prognostic circulating marker and potential therapeutic target in patients with tumor diseases. Curr Drug Targets.

