# Supplementary material for: Upregulation of the long non-coding RNA CASC9 as a biomarker for squamous cell carcinoma
Source: BMC Cancer. 2019 Aug 14;19:806. doi: 10.1186/s12885-019-6021-6 (PMC6694542; doi:10.1186/s12885-019-6021-6)
Supplement: Supplementary file 1 — Table S1. Oligonucleotide sequences for named targets. Forward and reverse primer sequences are given in 5′ to 3’orientation. Table S2. Strongly overexpressed lncRNAs in HNSCC tissues with potential prognostic value according to TCGA data. Table S3. Clinical and histopathological parameters of tissue set DUS. Table S4. Clinical and histopathological parameters of the TCGA cohort, * TNM, 4th–7th Edition. Table S5. Combined analysis of specificity and sensitivity for CASC9 and HOTAIR in the DUS HNSCC tissue sample set [37]. (DOC 255 kb) [file 12885_2019_6021_MOESM1_ESM.doc]

Table S1: Oligonucleotide sequences for named targets. Forward and reverse primer sequences are given in 5´ to 3´orientation

| **Target** | **Sequence** |
| --- | --- |
| **CASC9-qRT-f** | TTATGTTTGGCTGGAGAGT |
| **CASC9-qRT-r** | TTGCTTTGCTGCTGTCTG |
| **HOTAIR-qRT-f** | GGTAGAAAAAGCAACCACGAAGC |
| **HOTAIR-qRT-r** | ACATAAACCTCTGTCTGTGAGTGCC |
| **PDCD4-qRT-f** | AGTGACGCCCTTAGAAGTGG |
| **PDCD4-qRT-r** | TCATATCCACCTCCTCCACA |
| **CDK4-qRT-f** | ATGGCTACCTCTCGATATGAGC |
| **CDK4-qRT-r** | CATTGGGGACTCTCACACTCT |
| **CCND1-qRT-f** | TACTACCGCCTCACACGCTTC |
| **CCND1-qRT-r** | TTCGATCTGCTCCTGGCAG |
| **E-Cadherin-qRT-f** | ACCAGAATAAAGACCAAGTGACCA |
| **E-Cadherin-qRT-r** | AGCAAGAGCAGCAGAATCAGAAT |
| **BCL-2-qRT-f** | CCTGTGGATGACTGAGTACCTG |
| **BCL-2-qRT-r** | CAGAGGCCGCATGCTGGG |
| **TBP-qRT-f** | acaacagcctgccacctta |
| **TBP-qRT-r** | gaataggctgtggggtcagt |
| **GAPDH-qRT-f** | GAAGGTGAAGGTCGGAGTC |
| **GAPDH-qRT-r** | GAAGATGGTGATGGGATTTC |
| **SDHA-qRT-f** | GCCAGGACCTAGAGTTTGTTCA |
| **SDHA-qRT-r** | CTTTCGCCTTGACTGTTAATGA |

Table S2: Strongly overexpressed lncRNAs in HNSCC tissues with potential prognostic value according to TCGA data

| **gene name**  **UCSC genome browser** | **chromosomal localisation** | **size in bp** | **alternative name** | **neighbouring coding genes** | **lncRNA type** | **expression in HNSCC tissues** |
| --- | --- | --- | --- | --- | --- | --- |
| **ENSG00000231346.1** | 1p13.2 | 706 | LINC00160 | RAP1A | antisense | increased |
| **ENSG00000215808.2** | 1q43 | 1537 | LINC01139 | MTRNR2L11 | intragenic | increased |
| **ENSG00000229647.1** | 2q33.3 | 601 | Lnc-KLF7-1 | KLF7, MIR1302-4 | divergent | increased |
| **ENSG00000163364.5** | 2q31.1 | 838 | LINC01116 | MTX2 | intragenic | increased |
| **ENSG00000225548.1** | 3p24.1 | 491 | LINC01980 | EOMES | intragenic | increased |
| **ENSG00000237978.1** | 3q26.32 | 508 | KCNMB2-AS1 | KCNMB2 | antisense | increased |
| **ENSG00000248554.1** | 5p12 | 572 | Lnc-NNT2 | C5orf34 | divergent | increased |
| **ENSG00000223485.1** | 6q27 | 647b | LINC01615 | THBS2 | intragenic | increased |
| **ENSG00000254341.1** | 8q13.1 | 472 | SNHG6 | MCMDC2 | antisense | increased |
| **ENSG00000249395.2** | 8q21.11 | 1164 | **CASC9** | HNF4G | intragenic | increased |
| **ENSG00000242147.1** | 10p15.1 | 704 | Lnc-ASB13-3 | ASB13 | intragenic | increased |
| **ENSG00000254560.1** | 11p14.1 | 745 | BBOX1-AS1 | BBOX1 | antisense | increased |
| **ENSG00000249641.2** | 12q13.13 | 1408 | HOXC13-AS | HOXC13 | antisense | increased |
| **ENSG00000225210.5** | 14q11.2 | 1060 | Lnc-POTEM-4 | POTEM | intragenic | increased |
| **ENSG00000258955.1** | 14q22.1 | 1492 | LINC00640 | TMX1 | intragenic | increased |
| **ENSG00000272763.1** | 17q21.32 | 379 b | HOXB-AS4 | HOXB9  MIR196A1 | intragenic | increased |
| **ENSG00000237989.1** | 21q22.3 | 2798 b | LINC01679 | SIK1 | intragenic | increased |
| **ENSG00000206195.6** | 22q11.1 | 2102 b | Lnc-POTEH-7 | POTEH | pseudogene | increased |
| **ENSG00000273272.1** | 22q13.33 | 362 b | Lnc-KLHDC7B-2 | KLHDC7B | intragenic | increased |
| **ENSG00000272666.1** | 22q13.33 | 602 b | Lnc-SYCE3-1 | SYCE3 | intragenic | increased |

Table S3: Clinical and histopathological parameters of tissue set DUS

| **Variables** |  | **CASC9** |  |  | **HOTAIR** |  |  |
| --- | --- | --- | --- | --- | --- | --- | --- |
|  | n | median (range) | mean | p | median (range) | mean | p |
| **Age mean** |  | 64.78 y |  |  |  |  |  |
| **Age median** |  | 64.5 y |  |  |  |  |  |
| **Age range** |  | 53-90 y |  |  |  |  |  |
| **female** | 13 | 0.004 (0.0-0.447) | 0.069 | 0.99 | 0.010 (0.0-0.214) | 0.025 | 0.952 |
| **male** | 19 | 0.005 (0.0-0.557) | 0.07 |  | 0.008 (0.0-0.14) | 0.057 |  |
|  |  |  |  |  |  |  |  |
| **T stage pT1** | 6 | 0.079 (0.0-0.284) | 0.112 |  | 0.038 (0.001-0.235) | 0.088 |  |
| **T stage pT2** | 13 | 0.037 (0.001-0.557) | 0.143 |  | 0.010 (0.0-0.914) | 0.082 |  |
| **T stage pT3** | 6 | 0.006 (0.0-0.045) | 0.013 |  | 0.011 (0.003-0.046) | 0.017 |  |
| **T stage pT4** | 7 | 0.023 (0.0-0.255) | 0.065 |  | 0.011 (0.002-0.122) | 0.035 |  |
| **Tstage ≤ pT2** | 19 | 0.062 (0-0.557) | 0.133 | **0.041** | 0.016 (0.0-0.914) | 0.084 | 0.705 |
| **Tstage > pT2** | 13 | 0.008 (0.0-0.255) | 0.041 |  | 0.011 (0.002-0.122) | 0.026 |  |
|  |  |  |  |  |  |  |  |
| **Lymphnodes pos** | 17 | 0.024 (0.0-0.557) | 0.126 | 0.769 | 0.010 (0.0-0.914) | 0.083 | 0.83 |
| **Lymphnodes neg** | 15 | 0.030 (0.0-0.320) | 0.059 |  | 0.013 (0-0.214) | 0.037 |  |
| **HPV pos** | 6 | 0.006 (0.0-0.255) | 0.05 | 0.139 | 0.004 (0.0-0.021) | 0.007 | **0.023** |
| **HPV neg** | 20 | 0.053 (0.0-0.557) | 0.128 |  | 0.014 (0.0-0.914) | 0.085 |  |
|  |  |  |  |  |  |  |  |
| **dead** | 4 |  |  |  |  |  |  |
| **alive** | 29 |  |  |  |  |  |  |
| **T** | 32 | 0.030 (0.0-0.557) | 0.096 | **0.001** | 0.011 (0.0-0.914) | 0.061 | **0.001** |
| **N** | 12 | 0.0 (0.0-0.004) | 0.0008 |  | 0.0 (0.0-0.011) | 0.003 |  |

Table S4: Clinical and histopathological parameters of the TCGA cohort, * TNM, 4th-7th Edition

| **Variable** | **Number (n)** | **Mean CASC9 Expression [95%CI]** | **p-value; (Spearman’s *ρ*)** | **Mean HOTAIR Expression [95% CI]** | | **p-value; (Spearman’s *ρ*)** |  |  |
| --- | --- | --- | --- | --- | --- | --- | --- | --- |
| **All Patients** | 426 (100%) | 3.75 [3.41-4.09] |  | 0.158 [0.122-0.195] |  | |  |  |
|  |  |  |  |  |  | |  |  |
| **Age** | 425 (100%) |  | p=0.061; ***ρ***=0.091 |  | p=0.054; ***ρ***=0.094 | |  |  |
| **Mean** | 60.9 |  |  |  |  | |  |  |
| **Median** | 61.0 |  |  |  |  | |  |  |
| **Range** | 19-90 |  |  |  |  | |  |  |
|  |  |  |  |  |  | |  |  |
| **Gender** | 426 (100%) |  |  |  |  | |  |  |
| **Male** | 311 (73.0%) | 3.89 [3.49-4.29] |  | 0.139 [0.111-0.167] |  | |  |  |
| **Female** | 115 (27.0%) | 3.39 [2.78-3.99] | p=0.33 | 0.210 [0.096-0.324] | p=0.41 | |  |  |
|  |  |  |  |  |  | |  |  |
| **Smoking Category** | 416 (100%) |  |  |  |  | |  |  |
| **Lifelong Non-smoker** | 90 (21.6%) | 3.13 [2.44-3.83] |  | 0.142 [0.104-0.179] |  | |  |  |
| **Current Smoker** | 142 (34.1%) | 3.65 [3.12-4.18] |  | 0.187 [0.095-0.280] |  | |  |  |
| **Current reformed smoker for > 15 years** | 66 (15.9%) | 3.92 [2.95-4.88] |  | 0.124 [0.082-0.166] |  | |  |  |
| **Current reformed smoker for < or = 15 years** | 116 (27.9%) | 4.15 [3.49-4.80] |  | 0.159 [0.095-0.223] |  | |  |  |
| **Current Reformed Smoker. Duration Not Specified** | 2 (0.5%) | 0.90 [-10.6-12.5] | p=0.093 | 0.134 [-1.57-1.84] | p=0.79 | |  |  |
| **NA** | 10 |  |  |  |  | |  |  |
|  |  |  |  |  |  | |  |  |
| **Smoking (Pack Years)** | 243 (100%) |  | p=0.064; ***ρ***=0.119 |  | p=0.070; ***ρ***=-0.116 | |  |  |
| **Mean** | 47.1 |  |  |  |  | |  |  |
| **Median** | 40.0 |  |  |  |  | |  |  |
| **Range** | 0.02-300 |  |  |  |  | |  |  |
|  |  |  |  |  |  | |  |  |
| **Alcohol Consumption Frequency (Days per Week)** | 181 (100%) |  | p=0.80; ***ρ***=0.019 |  | p=0.15; ***ρ***=-0.107 | |  |  |
| **Mean** | 4.3 |  |  |  |  | |  |  |
| **Median** | 7.0 |  |  |  |  | |  |  |
| **Range** | 0-7 |  |  |  |  | |  |  |
|  |  |  |  |  |  | |  |  |
| **Daily Alcohol** | 178 (100%) |  | p=0.47; ***ρ***=-0.054 |  | **p=0.011; *ρ*=-0.190** | |  |  |
| **Mean** | 3.2 |  |  |  |  | |  |  |
| **Median** | 2.0 |  |  |  |  | |  |  |
| **Range** | 0-24 |  |  |  |  | |  |  |
|  |  |  |  |  |  | |  |  |
| **Location** |  |  |  |  |  | |  |  |
| **Oropharynx** | 61 (14.3%) | 4.24 [3.18-5.30] |  | 0.122 [0.074-0.171] |  | |  |  |
| **Oral Cavity** | 260 (61.0%) | 3.30 [2.88-3.72] |  | 0.155 [0.108-0.203] |  | |  |  |
| **Hypopharynx** | 6 (1.4) | 3.31 [0.43-6.19] |  | 0.162 [-0.086-0.410] |  | |  |  |
| **Larynx** | 99 (23.2%) | 4.67 [4.05-5.30] | **p<0.001** | 0.188 [0.094-0.281] | p=0.17 | |  |  |
|  |  |  |  |  |  | |  |  |
| **AJCC Stage (Clinical *)** | 413 (100%) |  |  |  |  | |  |  |
| **I** | 16 (3.9%) | 3.06 [1.43-4.68] |  | 0.107 [0.036-0.178] |  | |  |  |
| **II** | 86 (20.8%) | 3.29 [2.60-3.98] |  | 0.154 [0.081-0.227] |  | |  |  |
| **III** | 95 (23.0) | 3.81 [2.97-4.66] |  | 0.189 [0.065-0.313] |  | |  |  |
| **IV** | 216 (52.3) | 3.88 [3.44-4.32] | p=0.33 | 0.152 [0.114-0.191] | p=0.31 | |  |  |
| **NA** | 13 |  |  |  |  | |  |  |
|  |  |  |  |  |  | |  |  |
| **Clinical Tumor Category *** | 422 (100%) |  |  |  |  | |  |  |
| **cT1** | 27 (6.4%) | 2.87 [1.77-3.97] |  | 0.399 [-0.040-0.838] |  | |  |  |
| **cT2** | 128 (30.3%) | 3.57 [2.90-4.24] |  | 0.166 [0.092-0.239] |  | |  |  |
| **cT3** | 117 (27.7%) | 3.90 [3.23-4.56] |  | 0.135 [0.103-0.167] |  | |  |  |
| **cT4** | 139 (32.9%) | 3.88 [3.35-4.42] |  | 0.127 [0.102-0.152] |  | |  |  |
| **cTX** | 11 (2.6%) | 4.04 [1.94-6.14] | p=0.41 | 0.140 [0.067-0.213] | p=0.68 | |  |  |
| **NA** | 4 |  |  |  |  | |  |  |
|  |  |  |  |  |  | |  |  |
| **Clinical Nodal Category *** | 426 (100%) |  |  |  |  | |  |  |
| **cN0** | 202 (47.4%) | 3.63 [3.19-4.06] |  | 0.138 [0.103-0.173] |  | |  |  |
| **cN1** | 70 (16.4%) | 3.63 [2.64-4.62] |  | 0.205 [0.037-0.372] |  | |  |  |
| **cN2** | 127 (29.8%) | 3.78 [3.14-4.42] |  | 0.173 [0.111-0.235] |  | |  |  |
| **cN3** | 8 (1.9%) | 5.73 [3.92-7.55] |  | 0.133 0.029-0.238] |  | |  |  |
| **cNX** | 19 (4.5%) | 4.52 [2.46-6.58] | p=0.15 | 0.111 [0.074-0.147] | p=0.21 | |  |  |
|  |  |  |  |  |  | |  |  |
| **Clinical Distant Metastasis Category *** | 421 (100%) |  |  |  |  | |  |  |
| **cM0** | 407 (96.7%) | 3.72 [3.38-4.06] |  | 0.150 [0.115-0.184] |  | |  |  |
| **cM1** | 3 (0.7%) | 3.16 [-3.14-9.46] |  | 0.095 [0.008-0.183] |  | |  |  |
| **cMX** | 11 (2.6) | 3.96 [1.85-6.06] | p=0.89 | 0.510 [-0.174-1.19] | p=0.15 | |  |  |
| **NA** | 5 |  |  |  |  | |  |  |
|  |  |  |  |  |  | |  |  |
| **AJCC Stage *** | 364 (100%) |  |  |  |  | |  |  |
| **I** | 24 (6.6%) | 2.27 [1.06-3.48] |  | 0.307 [-0.103-0.717] |  | |  |  |
| **II** | 66 (18.1%) | 3.08 [2.36-3.79] |  | 0.156 [0.060-0.252] |  | |  |  |
| **III** | 73 (20.1%) | 4.31 [3.38-5.24] |  | 0.170 [0.075-0.266] |  | |  |  |
| **IV** | 201 (55.2%) | 3.89 [3.40-4.39] | **p=0.034** | 0.145 [0.105-0.185] | p=0.67 | |  |  |
| **NA** | 62 |  |  |  |  | |  |  |
|  |  |  |  |  |  | |  |  |
| **Tumor Category *** | 408 (100%) |  |  |  |  | |  |  |
| **T0** | 1 (0.2%) | 3.52 |  | 0.000 |  | |  |  |
| **T1** | 43 (10.5%) | 3.06 [1.79-4.33] |  | 0.285 [0.012-0.557] |  | |  |  |
| **T2** | 111 (27.2%) | 3.69 [2.98-4.41] |  | 0.178 [0.093-0.262] |  | |  |  |
| **T3** | 83 (20.3%) | 4.08 [3.38-4.78] |  | 0.125 [0.095-0.155] |  | |  |  |
| **T4** | 133 (32.6%) | 3.84 [3.26-4.42] |  | 0.129 [0.103-0.155] |  | |  |  |
| **TX** | 37 (9.1%) | 3.87 [2.66-5.07] | p=0.20 | 0.152 [0.082-0.221] | p=0.65 | |  |  |
| **NA** | 18 |  |  |  |  | |  |  |
|  |  |  |  |  |  | |  |  |
| **Nodal Category *** | 407 (100%) |  |  |  |  | |  |  |
| **N0** | 146 (35.9%) | 3.49 [3.00-3.97] |  | 0.176 [0.096-0.255] |  | |  |  |
| **N1** | 57 (14.0%) | 3.96 [2.79-5.14] |  | 0.178 [0.055-0.301] |  | |  |  |
| **N2** | 127 (31.2%) | 3.83 [3.20-4.47] |  | 0.152 [0.093-0.210] |  | |  |  |
| **N3** | 6 (1.5%) | 5.42 [0.48-10.4] |  | 0.212 [0.038-0.385] |  | |  |  |
| **NX** | 71 (17.4%) | 3.91 [3.03-4.79] | p=0.90 | 0.122 [0.082-0.161] | p=0.43 | |  |  |
| **NA** | 19 |  |  |  |  | |  |  |
|  |  |  |  |  |  | |  |  |
| **Distant Metastasis Category** | 167 (100%) |  |  |  |  | |  |  |
| **M0** | 138 (82.6%) | 3.82 [3.21-4.42] |  | 0.132 [0.080-0.184] |  | |  |  |
| **M1** | 0 (0%) | NA |  | NA |  | |  |  |
| **MX** | 29 (17.4%) | 3.39 [1.94-4.83] | p=0.38 | 0.307 [0.054-0.561] | p=0.097 | |  |  |
| **NA** | 259 |  |  |  |  | |  |  |
|  |  |  |  |  |  | |  |  |
| **Histologic Grade** | 425 (100%) |  |  |  |  | |  |  |
| **G1** | 52 (12.2%) | 3.49 [2.43-4.56] |  | 0.095 [0.069-0.121] |  | |  |  |
| **G2** | 250 (58.8) | 3.66 [3.25-4.08] |  | 0.134 [0.095-0.173] |  | |  |  |
| **G3** | 104 (24.5%) | 3.96 [3.23-4.68] |  | 0.249 [0.134-0.364] |  | |  |  |
| **G4** | 7 (1.7%) | 3.60 [0.52-6.69] |  | 0.067 [-0.010-0.143} |  | |  |  |
| **GX** | 12 (2.8%) | 5.26 [2.50-8.02] | p=0.71 | 0.212 [0.060-0.363] | **p=0.002** | |  |  |
| **NA** | 1 |  |  |  |  | |  |  |
|  |  |  |  |  |  | |  |  |
| **Surgical Margin** | 371 (100%) |  |  |  |  | |  |  |
| **Positive** | 42 (11.3%) | 4.46 [3.38-5.54] |  | 0.276 [0.046-0.506] |  | |  |  |
| **Close** | 37 (10.0%) | 3.61 [2.16-5.06] |  | 0.245 [-0.018-0.508] |  | |  |  |
| **Negative** | 292 (78.7%) | 3.60 [3.20-4.00] | p=0.13 | 0.137 [0.110-0.164] | p=0.96 | |  |  |
| **NA** | 55 |  |  |  |  | |  |  |
|  |  |  |  |  |  | |  |  |
| **Extracapsular Spread (Pathologic)** | 279 (100%) |  |  |  |  | |  |  |
| **No** | 196 (70.3%) | 3.23 [2.77-3.69] |  | 0.169 [0.100-0.238] |  | |  |  |
| **Microscopic** | 56 (20.1%) | 4.12 [3.14-5.10] |  | 0.205 [0.078-0.333] |  | |  |  |
| **Gross** | 27 (9.7%) | 5.85 [3.80-7.90] | **p=0.020** | 0.163 [0.086-0.241] | p=0.11 | |  |  |
| **NA** | 147 |  |  |  |  | |  |  |
|  |  |  |  |  |  | |  |  |
| **Angiolymphatic Invasion** | 276 (100%) |  |  |  |  | |  |  |
| **Yes** | 90 (32.6%) | 4.12 [3.27-4.98] |  | 0.205 [0.096-0.315] |  | |  |  |
| **No** | 186 (67.4%) | 3.53 [3.06-4.00] | p=0.61 | 0.138 [0.100-0.177] | p=0.52 | |  |  |
| **NA** | 150 |  |  |  |  | |  |  |
|  |  |  |  |  |  | |  |  |
| **HPV Status** | 279 (100%) |  |  |  |  | |  |  |
| **Positive** | 36 (12.9%) | 3.61 [2.49-4.72] |  | 0.108 [0.051-0.165] |  | |  |  |
| **Negative** | 243 (87.1%) | 3.80 [3.37-4.23] | p=0.79 | 0.130 [0.101-0.160] | p=0.16 | |  |  |
| **NA** | 147 |  |  |  |  | |  |  |
|  |  |  |  |  |  | |  |  |
| **Mutation Count** | 279 (100%) |  | **p<0.001; *ρ*=0.213** |  | p=0.72; ***ρ***=0.022 | |  |  |
| **Mean** | 138 |  |  |  |  | |  |  |
| **Median** | 102 |  |  |  |  | |  |  |
| **Range** | 3-1.422 |  |  |  |  | |  |  |
|  |  |  |  |  |  | |  |  |
| **Copy Number Alterations** | 422 (100%) |  | **p<0.001; *ρ*=0.270** |  | **p=0.003; *ρ*=0.142** | |  |  |
| **Mean** | 0.24 |  |  |  |  | |  |  |
| **Median** | 0.23 |  |  |  |  | |  |  |
| **Range** | 0-0.89 |  |  |  |  | |  |  |

Table S5: Combined analysis of specificity and sensitivity for CASC9 and HOTAIR in the DUS HNSCC tissue sample set

| Expression of both lncRNAs | T | N |
| --- | --- | --- |
| Higher than respective ROC threshold | A:19 | B:0 |
| Equal or lower than respective ROC threshold | C:20 | D:14 |

Specificity = D/B+D =1.0 Sensitivity = A/A+C =0.48

Calculated according to Cancer Epidemiology: Principles and Methods (WHO).
