# Supplementary figures and images for: Upregulation of the long non-coding RNA CASC9 as a biomarker for squamous cell carcinoma
Source: BMC Cancer. 2019 Aug 14;19:806. doi: 10.1186/s12885-019-6021-6 (PMC6694542; doi:10.1186/s12885-019-6021-6)

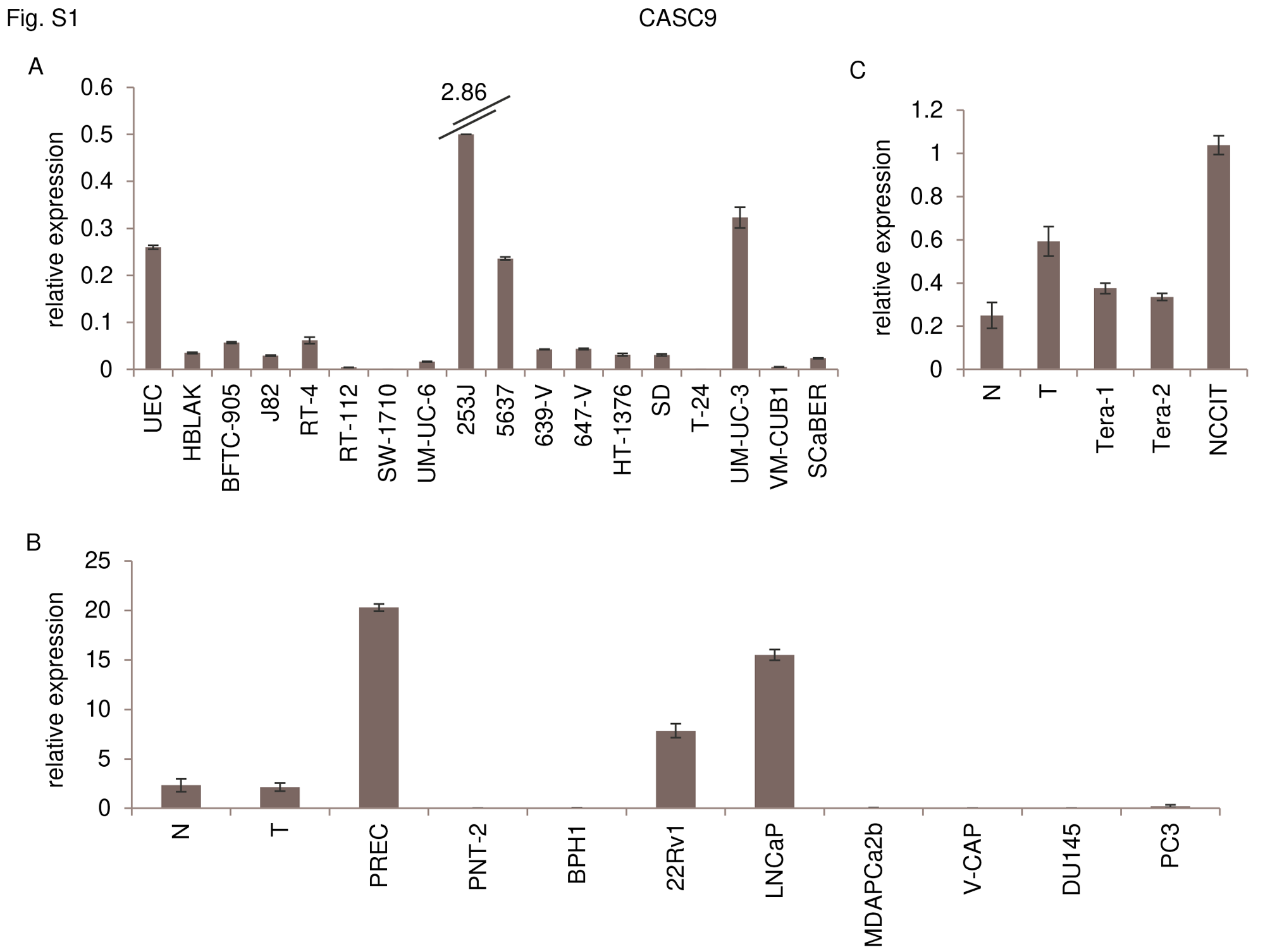

Supplement: Supplementary file 2 — Figure S1. Expression of CASC9 in cell lines of other tumor entities. (a) Relative expression of CASC9 was determined by RT-qPCR across 16 urothelial carcinoma cell lines compared to the benign urothelial control cell line HBLAK and a primary urothelial cell culture (UEC). (b) Relative expression of CASC9 in prostate cancer cell lines, benign control cells (PREC, PNT-2, BPH-1) as well as normal (N) and cancerous (T) tissue. (c) Relative expression of CASC9 in testicular cancer cell lines compared to normal (N) and cancerous (T) tissue. Figure S2. Expression of a putative downstream target gene PDCD4 in HNSCC and UCC cell lines. (a) Relative expression of PDCD4 mRNA was determined by RT-qPCR across 21 HNSCC cell lines compared to benign HaCat cells. (b) Relative PDCD4 expression across 16 urothelial carcinoma cell lines compared to the benign urothelial control cell line HBLAK and a primary urothelial cell culture (UEC). (c) Relative expression of PDCD4 in cells with CASC9 overexpression or downregulation (sh). No significant changes were observed. Figure S3. Expression of a putative downstream target genes CDK4, CCND1, CDH1 and BCL2 in HNSCC cell lines. (a) Relative expression of CDK4 mRNA was determined by RT-qPCR in cell lines following CASC9 modulation and across 21 HNSCC cell lines compared to benign HaCat cells. (b) Relative expression of CCND1 mRNA was determined by RT-qPCR in cell lines following CASC9 modulation and across 21 HNSCC cell lines compared to benign HaCat cells. (c) Relative expression of E-Cadherin mRNA was determined by RT-qPCR in cell lines following CASC9 modulation and across 21 HNSCC cell lines compared to benign HaCat cells. (d) Relative expression of BCL2 mRNA was determined by RT-qPCR in cell lines following CASC9 modulation and across 21 HNSCC cell lines compared to benign HaCat cells. (ZIP 1403 kb) [file 12885_2019_6021_MOESM2_ESM.zip › FigS1R2.tif]

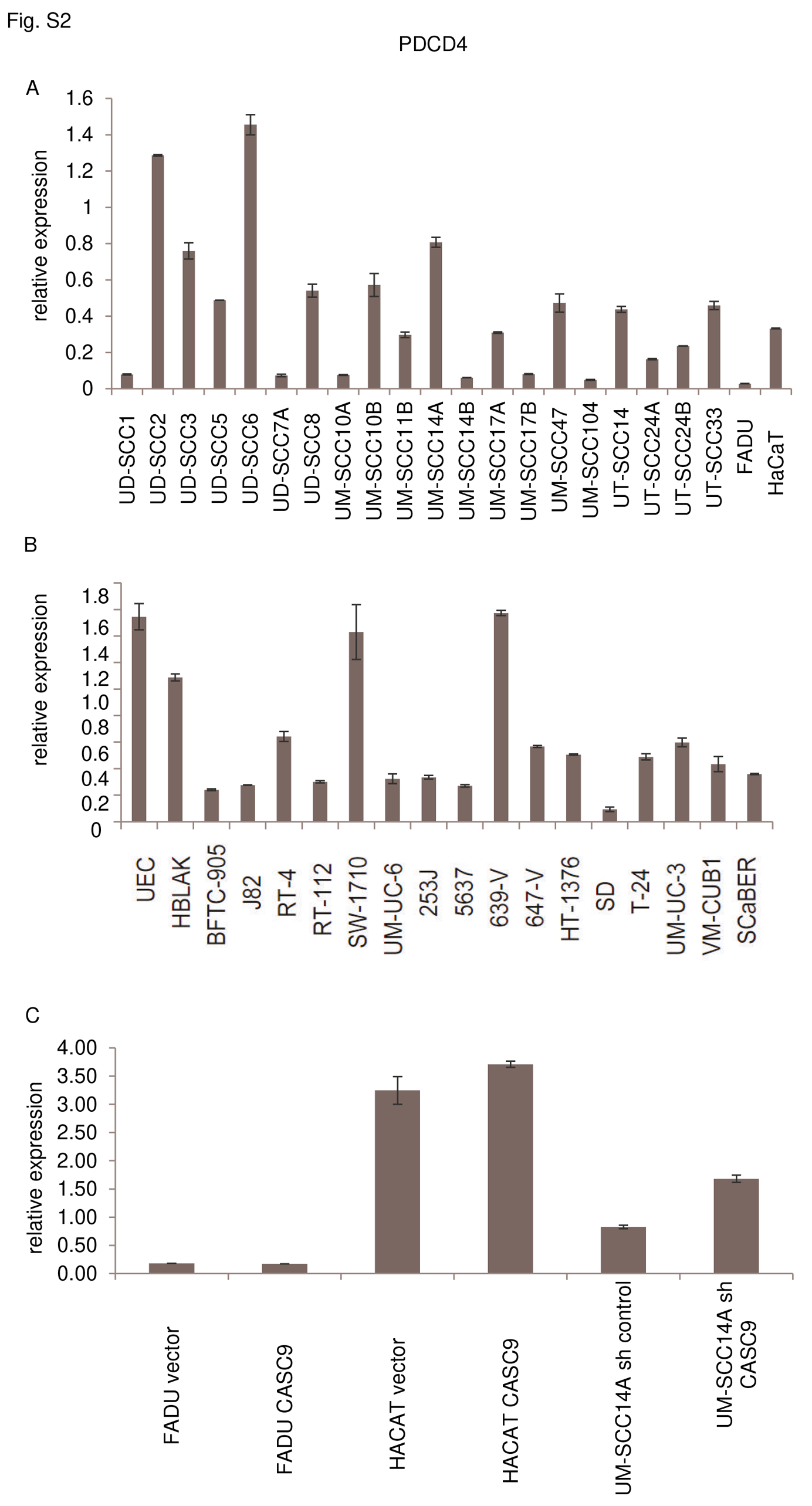

Supplement: Supplementary file 2 — Figure S1. Expression of CASC9 in cell lines of other tumor entities. (a) Relative expression of CASC9 was determined by RT-qPCR across 16 urothelial carcinoma cell lines compared to the benign urothelial control cell line HBLAK and a primary urothelial cell culture (UEC). (b) Relative expression of CASC9 in prostate cancer cell lines, benign control cells (PREC, PNT-2, BPH-1) as well as normal (N) and cancerous (T) tissue. (c) Relative expression of CASC9 in testicular cancer cell lines compared to normal (N) and cancerous (T) tissue. Figure S2. Expression of a putative downstream target gene PDCD4 in HNSCC and UCC cell lines. (a) Relative expression of PDCD4 mRNA was determined by RT-qPCR across 21 HNSCC cell lines compared to benign HaCat cells. (b) Relative PDCD4 expression across 16 urothelial carcinoma cell lines compared to the benign urothelial control cell line HBLAK and a primary urothelial cell culture (UEC). (c) Relative expression of PDCD4 in cells with CASC9 overexpression or downregulation (sh). No significant changes were observed. Figure S3. Expression of a putative downstream target genes CDK4, CCND1, CDH1 and BCL2 in HNSCC cell lines. (a) Relative expression of CDK4 mRNA was determined by RT-qPCR in cell lines following CASC9 modulation and across 21 HNSCC cell lines compared to benign HaCat cells. (b) Relative expression of CCND1 mRNA was determined by RT-qPCR in cell lines following CASC9 modulation and across 21 HNSCC cell lines compared to benign HaCat cells. (c) Relative expression of E-Cadherin mRNA was determined by RT-qPCR in cell lines following CASC9 modulation and across 21 HNSCC cell lines compared to benign HaCat cells. (d) Relative expression of BCL2 mRNA was determined by RT-qPCR in cell lines following CASC9 modulation and across 21 HNSCC cell lines compared to benign HaCat cells. (ZIP 1403 kb) [file 12885_2019_6021_MOESM2_ESM.zip › FigS2R2.tif]

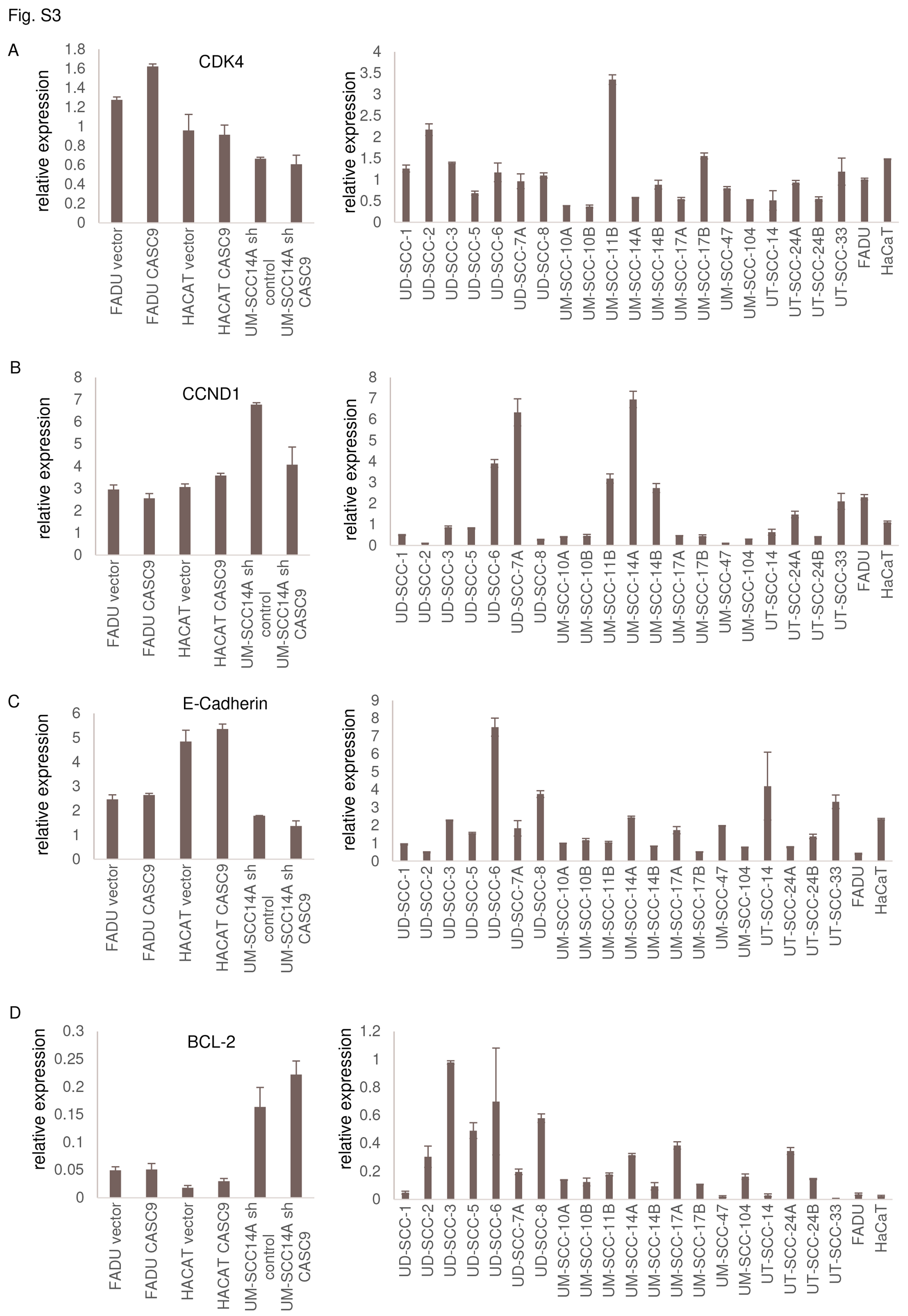

Supplement: Supplementary file 2 — Figure S1. Expression of CASC9 in cell lines of other tumor entities. (a) Relative expression of CASC9 was determined by RT-qPCR across 16 urothelial carcinoma cell lines compared to the benign urothelial control cell line HBLAK and a primary urothelial cell culture (UEC). (b) Relative expression of CASC9 in prostate cancer cell lines, benign control cells (PREC, PNT-2, BPH-1) as well as normal (N) and cancerous (T) tissue. (c) Relative expression of CASC9 in testicular cancer cell lines compared to normal (N) and cancerous (T) tissue. Figure S2. Expression of a putative downstream target gene PDCD4 in HNSCC and UCC cell lines. (a) Relative expression of PDCD4 mRNA was determined by RT-qPCR across 21 HNSCC cell lines compared to benign HaCat cells. (b) Relative PDCD4 expression across 16 urothelial carcinoma cell lines compared to the benign urothelial control cell line HBLAK and a primary urothelial cell culture (UEC). (c) Relative expression of PDCD4 in cells with CASC9 overexpression or downregulation (sh). No significant changes were observed. Figure S3. Expression of a putative downstream target genes CDK4, CCND1, CDH1 and BCL2 in HNSCC cell lines. (a) Relative expression of CDK4 mRNA was determined by RT-qPCR in cell lines following CASC9 modulation and across 21 HNSCC cell lines compared to benign HaCat cells. (b) Relative expression of CCND1 mRNA was determined by RT-qPCR in cell lines following CASC9 modulation and across 21 HNSCC cell lines compared to benign HaCat cells. (c) Relative expression of E-Cadherin mRNA was determined by RT-qPCR in cell lines following CASC9 modulation and across 21 HNSCC cell lines compared to benign HaCat cells. (d) Relative expression of BCL2 mRNA was determined by RT-qPCR in cell lines following CASC9 modulation and across 21 HNSCC cell lines compared to benign HaCat cells. (ZIP 1403 kb) [file 12885_2019_6021_MOESM2_ESM.zip › FigS3R2.tif]
